# Very long-/ and long Chain-3-Hydroxy Acyl CoA Dehydrogenase Deficiency correlates with deregulation of the mitochondrial fusion/fission machinery

**DOI:** 10.1038/s41598-018-21519-2

**Published:** 2018-02-19

**Authors:** Judith Hagenbuchner, Sabine Scholl-Buergi, Daniela Karall, Michael J. Ausserlechner

**Affiliations:** 1 Department of Pediatrics II, Medical University Innsbruck, Innsbruck, Austria; 2 Department of Pediatrics I, Medical University Innsbruck, Innsbruck, Austria

## Abstract

Children diagnosed with Long-Chain-3-Hydroxy-Acyl-CoA-Dehydrogenase-Deficiency (LCHADD) or Very-Long-Chain-3-Hydroxy-Acyl-CoA-Dehydrogenase-Deficiency **(**VLCADD) frequently present with hypertrophic cardiomyopathy or muscle weakness which is caused by the accumulation of fatty acid metabolites due to inactivating mutations in the mitochondrial trifunctional protein. By analyzing mitochondrial morphology we uncovered that mutations within the *HADHA* or the *ACADVL* gene not only affect fatty acid oxidation, but also cause significant changes in the DNM1L/MFN2 ratio leading to the significant accumulation of truncated and punctate mitochondria in contrast to network-like mitochondrial morphology in controls. These striking morphological abnormalities correlate with changes in OXPHOS, an imbalance in ROS levels, reduced mitochondrial respiration, reduced growth rates and significantly increased glucose uptake per cell, suggesting that *HADHA* and *ACADVL* mutations shift cellular energy household into glycolysis. Experiments using the NOX2-specific inhibitor Phox-I2 suggest that NOX2 is activated by accumulating long-chain fatty acids and generates ROS, which in turn changes mitochondrial morphology and activity. We thereby provide novel insights into the cellular energy household of cells from LCHADD/VLCADD patients and demonstrate for the first time a connection between fatty acid metabolism, mitochondrial morphology and ROS in patients with these rare genetic disorders.

## Introduction

Long-chain 3-hydroxyacyl-CoA dehydrogenase deficiency (LCHADD) (OMIM #609016) is an autosomal recessively inherited disorder of long-chain fatty acid oxidation with an estimated overall frequency of 1:50,000, first described in 1989 in children presenting with hypoketotic hypoglycemia and lethargy after periods of fasting, often associated with febrile infections and gastroenteritis^1,2^. Clinical symptoms mainly develop during episodes of illness or fasting and affect organs needing long-chain fat as primary energy source such as heart and skeletal muscle^3–5^.

The enzyme HADHA is part of the mitochondrial trifunctional protein (MTP) and specific for the metabolism of C12-C16 chain-length fatty acid compounds. Mutations of this protein leads to an accumulation of toxic β-oxidation intermediates causing immediate symptoms (hypoketotic hypoglycemia, hypertrophic cardiomyopathy, congestive heart failure, hepatomegaly and muscle weakness) as well as long-term complications, such as retinopathy and polyneuropathy^3,4^. From the long-chain fatty acid oxidation disorders, these long term complications are only seen in LCHADD patients, and the underlying pathophysiology on the cellular level remain unclear and not understood.

Beside their involvement in fatty acid oxidation mitochondria are in the center of cell death regulation and energy production. Most ATP is produced by oxidative phosphorylation, a mechanism which is tightly linked to the connectivity of the mitochondria. Beside members of the BCL2 family which mainly affect mitochondria during apoptosis induction^6,7^, mitochondrial morphology is regulated by the family of fusion/fission proteins. In mammals, mitochondrial fusion/fission dynamics are controlled by dynamin-1-like (DNM1L/DRP1) which promotes fragmentation of mitochondria, whereas mitofusion-1 (MFN1) and mitofusion-2 (MFN2) as well as optic-atrophy-1 (OPA1) induce mitochondrial fusion of the outer membrane and the inner mitochondrial membranes, respectively^8^.

Earlier papers on LCHADD patients have reported reduced mitochondrial activity in fibroblasts from LCHADD patients^9,10^. Here we demonstrate for the first time that skin fibroblasts from LCHADD and VLCADD patients show a significantly altered mitochondrial morphology and increased ROS levels. This suggests a deregulation of mitochondrial fusion/fission dynamics which correlated with a marked deregulation of the MFN2/DNM1L ratio, significantly impaired basal mitochondrial respiration and mitochondrial spare capacity, which in turn shifts energy production into glycolysis.

## Results

### Alteration of mitochondrial morphology in LCHADD/VLCADD patients

**Figure 1:**
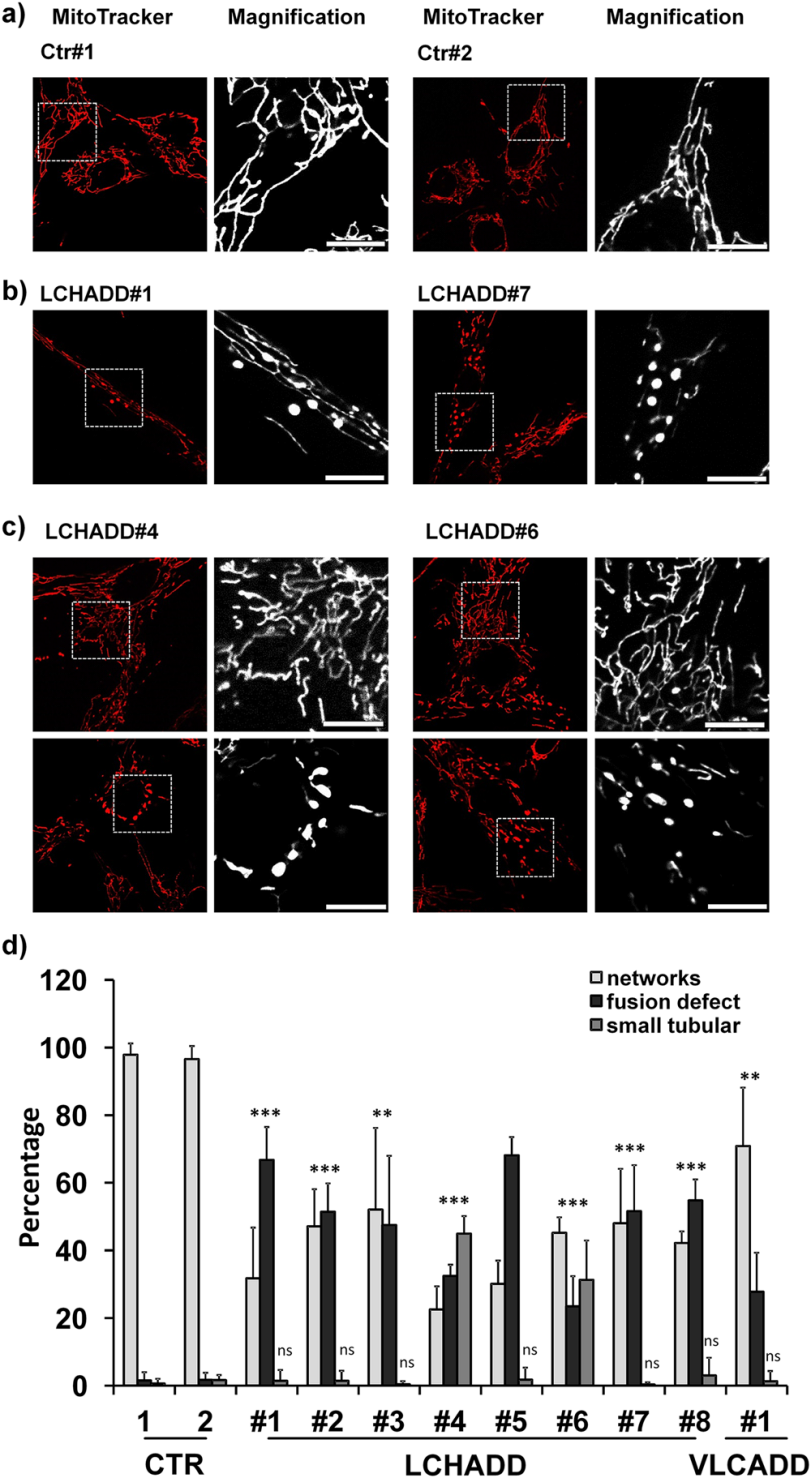
LCHADD/VLCADD affect mitochondrial morphology. Mitochondrial morphology was analyzed in fibroblasts of healthy controls (**a**) or LCHADD/VLCADD patients (n = 9; **b**,**c**). Mitochondrial morphology was classified into networks-like, fusion defect, or small tubular structure. (**d**) Shown is the mean of three independent experiments; for each experiment 30–40 cells were analyzed. Mitochondrial morphology classes were compared between control cells and patient cells (**p < 0.01; ***p < 0.001).

Clinical symptoms of LCHADD/VLCADD mainly occur during fasting, illness or physical strain and thereby affect organs with high energy consumption like the heart and the skeletal muscle. Since mitochondria are in the center of cellular energy generation which is linked to their connectivity^6^, we analyzed mitochondrial morphology *via* live cell imaging. 9/9 patient-derived skin fibroblast cultures showed alterations in their mitochondrial structures compared to healthy controls (Fig. 1a–d). Patient cells could be divided into mainly dot-like structures (Fig. 1b, 7/9 patients) and patients with mixed forms of dot-like structures and lots of very small tubes (small tubular, Fig. 1c, 2/9) compared to two healthy controls, which possessed more than 95% of network-like mitochondria (Fig. 1a,d).

**Figure 2:**
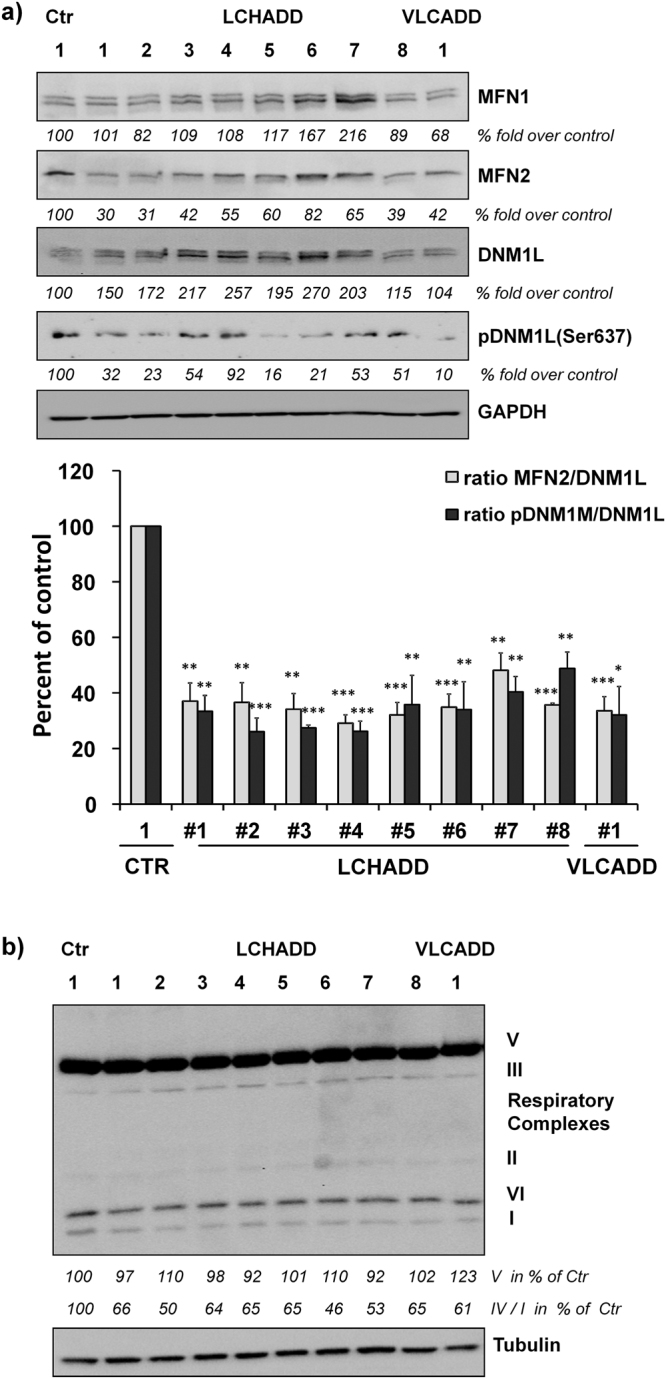
MFN2/DNM1L ratio is decreased in patients with LCHADD/VLCADD. Total protein of fibroblasts from healthy control or from patients with LCHADD or VLCADD was analyzed for expression of (**a**) MFN1, MFN2, DNM1L, pDNM1L, and GAPDH (loading control) or (**b**) OXPHOS and α-Tubulin by immunoblot. Samples were derived from the same experiment and gels/blots were processed in parallel. Densitometric analyses were performed using Labworks software version 4.5 (UVP, UK) and expressed as fold over healthy control. The ratio between MFN2/DNM1L and between pDNM1L/DNM1L was assessed from three independent experiments (*p < 0.05; **p < 0.01; ***p < 0.001). Uncropped immunoblots from three independent experiments are provided in Supplemental Fig. 1.

**Figure 3:**
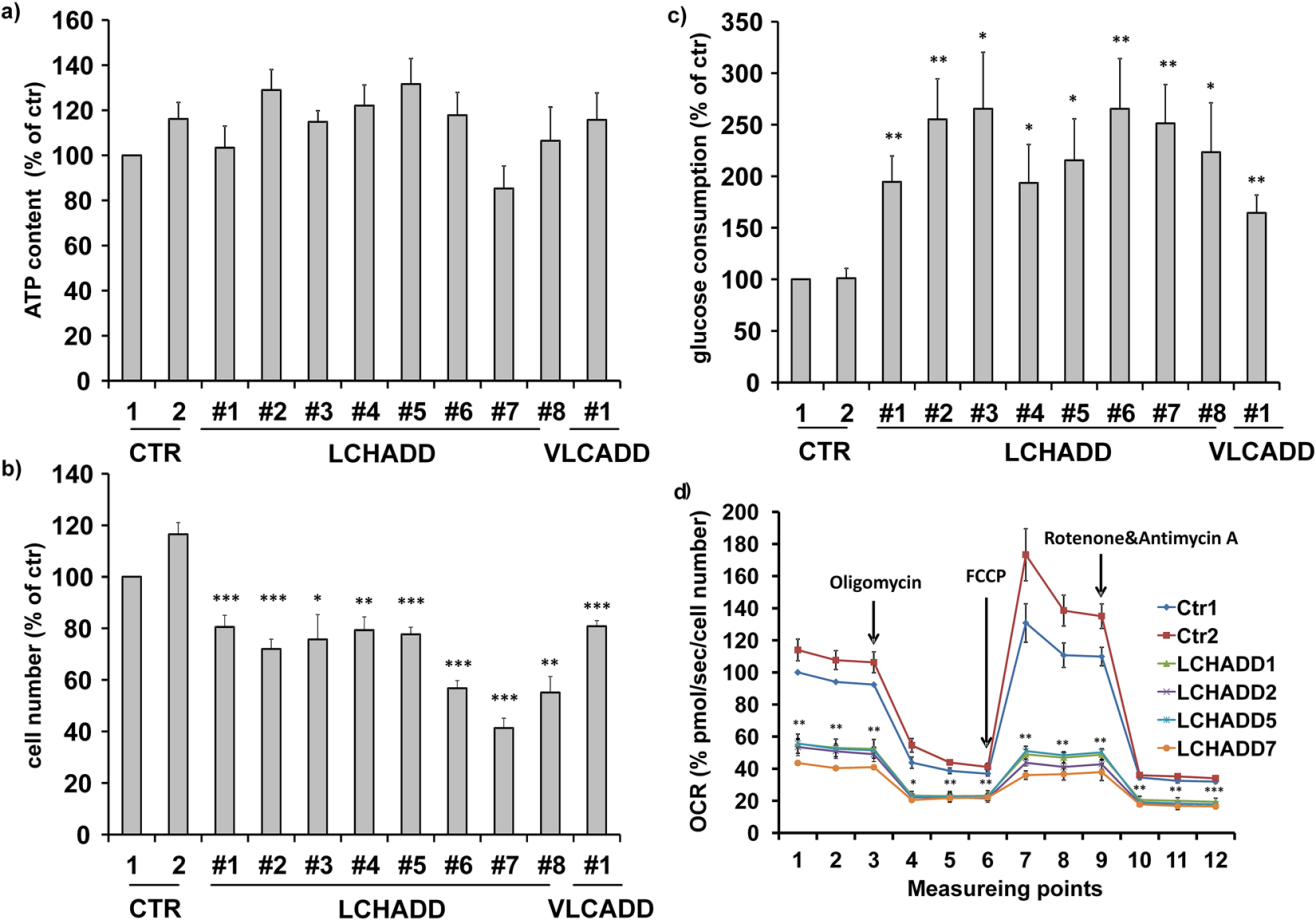
LCHADD fibroblasts exhibit increased aerobic glycolysis and impaired mitochondrial respiration. ATP amount (**a**), cell number (**b**) and glucose consumption (**c**) were measured in fibroblasts from two healthy controls, eight LCHADD patients, and one VLCADD patient. Shown is the mean ± s.e.m of four (**a**) or five (**b,c**) independent experiments (*p < 0.05; **p < 0.01; ***p < 0.001). (**d**) Mitochondrial respiration (Oxygen consumption rate, OCR) was measured in selected LCHADD patient fibroblasts and healthy controls using an Agilent Seahorse XFp Cell mito stress test kit. Arrows indicate the injection points of Oligomycin (1 µM), FCCP (0.5 µM) and Rotenone/Antimycin A (0.5 µM each). Shown is the mean of four independent experiments. Each patient-derived cell line was compared to each control. The lowest significance of each comparison is indicated (*p < 0.05; **p < 0.01) with all other comparisons being of higher significance.

Mitochondrial morphology in viable cells is mainly controlled by members of the fusion/fission family. Therefore we quantified the amounts of the main fusion proteins MFN1 and MFN2 as well as the mammalian regulator of fission DNM1L by immunoblot analyses. MFN1 differed compared to controls with 2 of 9 patients showing increased levels (patient #6 and #7), whereas others show no regulation (4/9) or decreased levels (3/9). MFN2, however, was decreased in all patients compared to the healthy control, ranging from 82% (patient #6) to 30% (patient #1). Interestingly the expression of DNM1L, the main fission regulator, was also increased in 8/9 patient fibroblasts most prominently in those patients, whose MFN2 repression was only moderate (patient #4, #5, #6, #7). Therefore we calculated the ratio between MFN2 and DNM1L expression which was significantly reduced in all patients from 48% (LCHADD patient #7) to even 29% (patient #4) compared to controls (100%) (Fig. 2a). Since DNM1L is inactivated by phosphorylation at serine 637, we next assessed pDNM1L(Ser637) phosphorylation by phospho-specific immunoblot analyses. DNM1L(Ser637) phosphorylation was reduced in 9 of 9 patients and the ratio of pDNM1L to total DNM1L was lowered to 49% (patient #8) to even 26% (patient #2)). This suggests that the observed morphological changes of mitochondria in Fig. 1 result from a marked disequilibrium of fusion/fission dynamics in patients with LCHADD or VLCADD. Since mitochondrial morphology and connectivity influences the respiration efficacy, we further analyzed the expression of OXPHOS complexes by immunoblot. In line with the morphological changes, we also observed reduced levels of complex I and IV, whereas complex II, III, and complex V, the ATP synthase, were not regulated compared to a healthy control (Fig. 2b). To further test, how these changes affect the metabolic activity and energy metabolism in patient fibroblasts, we analyzed the ATP amount in fibroblasts from healthy controls and from LCHADD and VLCADD patients. Although our immunoblots suggest reduced activity of the respiratory chain complexes I and IV, 8 of 9 fibroblasts from patients showed slightly, although not significantly, increased ATP content (Fig. 3a). Since ATP can also be generated *via* aerobic glycolysis, we further analyzed the glucose consumption by these skin fibroblasts. Interestingly, we observed a statistically significant reduction in cell growth in all patient-derived fibroblasts compared to the healthy controls (Fig. 3b) and a significantly increased glucose consumption *per* cell of LCHADD/VLCADD patients (Fig. 3c). To further investigate mitochondrial respiration in LCHADD fibroblasts in detail, we measured respiratory function using a Seahorse XFp System. The combined data of four different experiments revealed striking, highly significant differences between control fibroblast and four selected LCHADD patient fibroblasts: LCHADD fibroblasts had only 40–60% basal mitochondrial respiration compared to fibroblasts from healthy controls. Injection of oligomycin, an inhibitor of ATP-synthase, decreased respiration both in controls and LCHADD patients and this decrease reflects the production of ATP associated with mitochondrial activity – which is significantly lower in LCHADD patients than in controls. FCCP that uncouples ion transport through the inner mitochondrial membrane, leads to maximal O_2_-consumption by complex IV and is a measure for the maximal respiratory capacity. Whereas in controls FCCP increased O_2_-consumption to about 130–150% of basal respiration, in LCHADD cells FCCP did not elevate O_2_-consumption above basal respiration, suggesting that LCHADD patient-derived fibroblasts surprisingly have no respiratory spare capacity. This suggests that LCHADD patient-derived fibroblasts are already working on their maximum capacity and cannot compensate increased energy demand by increasing their mitochondrial respiration. Our results therefore demonstrate that LCHADD/VLCADD mutations not only hamper fatty acid oxidation, but that fibroblasts from such patients metabolize significantly more glucose to balance ATP generation despite impaired mitochondrial efficacy.

**Figure 4:**
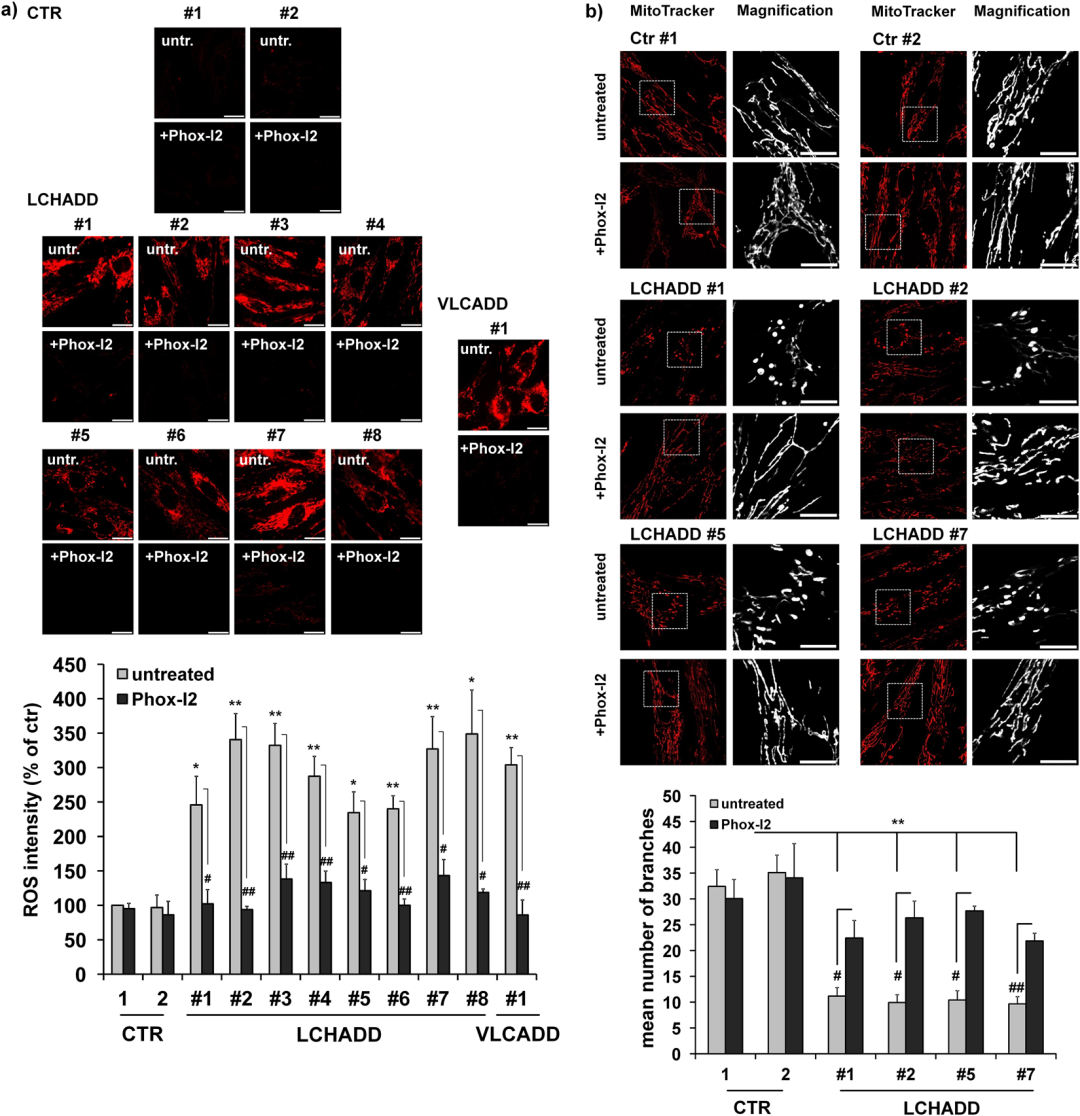
ROS accumulation by NOX2 in LCHADD/VLCADD patient fibroblasts triggers mitochondrial fission. ROS levels in LCHADD/VLCADD patients and two healthy controls were analyzed by CM-H_2_XROS staining (500 nM) with or without Phox-I2 (15 µM, 24 hours added prior to analyses) (**a**). Images were acquired on an Axiovert200M microscope, equipped with a 63x oil objective. Bar size is 10 µm. Shown are representative images and densitometric quantification from three independent experiments. Significantly different between healthy controls and LCHADD/VLCADD patients: *p < 0.05; **p < 0.01. Significantly different between untreated and Phox-I2 treatment: ^#^p < 0.05; ^##^p < 0.01. Mitochondrial morphology was analyzed after 24 hours of Phox-I2 (15 µM) treatment in two healthy control fibroblast lines and LCHADD patient fibroblasts #1, #2 #5 and #7 (**b**). Bar size is 10 µm. For quantification at least thirty 30 µm × 30 µm images from three independent experiments were analyzed for the number of mitochondrial branches. Shown is the mean of three independent experiments. Significantly different between healthy controls and LCHADD patients: **p < 0.01. Significantly different between untreated cells and Phox-I2 treament: ^#^p < 0.05; ^##^p < 0.01.

**Figure 5:**
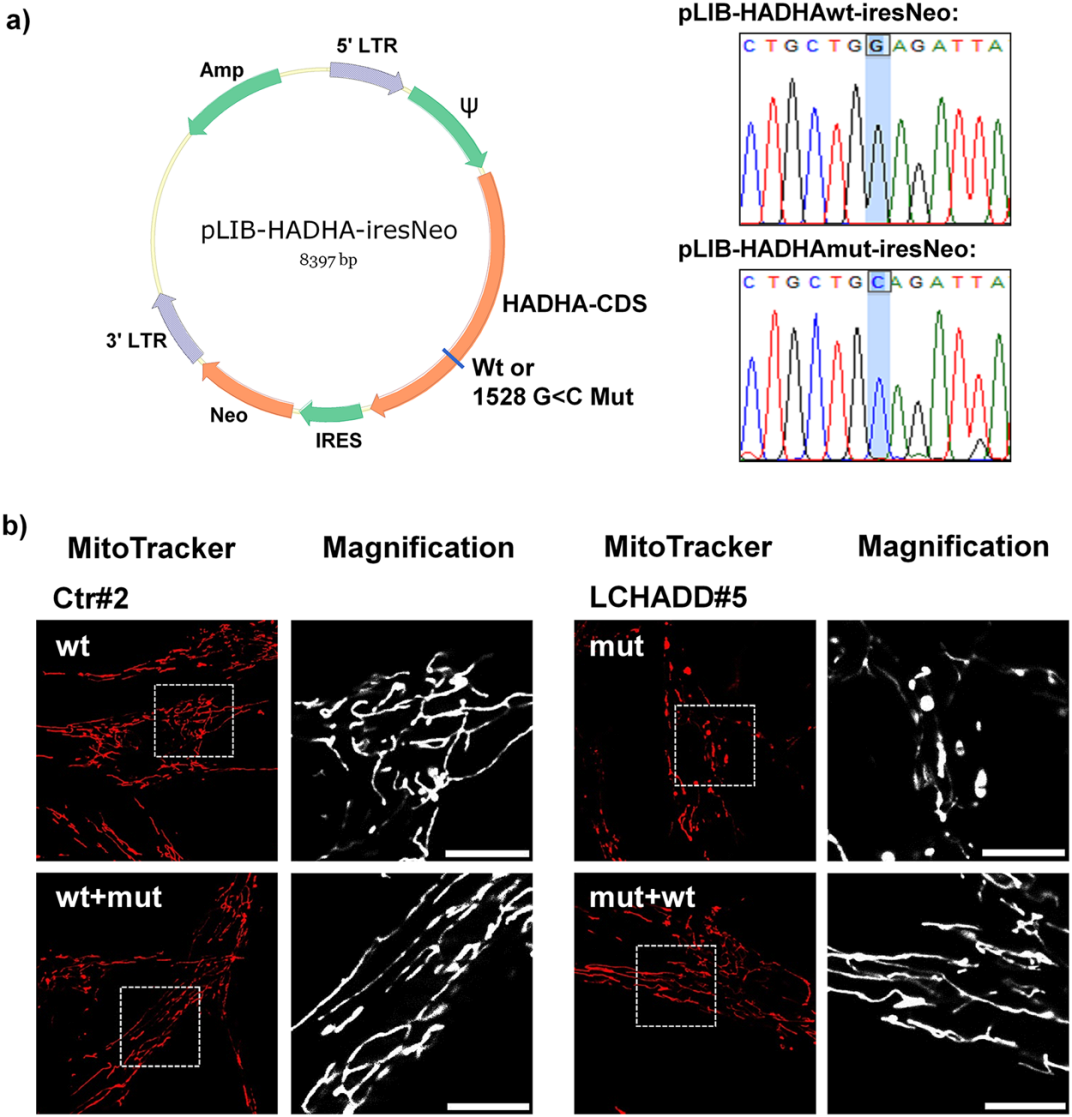
Expression of wild-type HADHA in LCHADD patient-derived fibroblasts induces mitochondrial elongation. The cDNA for wild-type HADHA and HADHAmut (1528 G < C) was amplified from control fibroblasts and patient #5, cloned into the retroviral vector pLIB-MCS-iresNeo and sequenced (**a**). The patient derived fibroblast cell line FB-LCHADD5-HADHAwt and FB-Ctr2-HADHAmut were generated by retrovial infection and neomycin selection. Mitochondrial morphology was analyzed by CMXRos staining (300 nM). Shown are representative images of controls and HADHAwt or HADHAmut overexpressing cells. Bar size is 10 µm.

One trigger of DNM1L-activation and mitochondrial fission is the accumulation of mitochondrial reactive oxygen species (ROS) as fission can be seen as a mechanism to isolate defective, ROS-producing mitochondria from the mitochondrial network. Interestingly, fibroblasts from all patients showed significantly increased steady state levels of ROS compared to healthy control fibroblasts (Fig. 4a). A recent publication implicated the NADPH-oxidase-2 (NOX2) as a source of ROS in palmitate-treated rodent beta cells^11^. The treatment with 15 µM of the NOX2-inhibitor Phox-I2 significantly reverted ROS-accumulation in all patients (Fig. 4) and led to re-fusion of mitochondrial networks (Fig. 4b). This suggest that NOX2-activity increases ROS levels, which in turn causes mitochondrial fission, reduced mitochondrial respiration and therefore increased glycolysis to compensate the energy demand. To finally prove whether the observed changes in mitochondrial morphology are caused by the homozygous mutation in the *HADHA* gene, we amplified the HADHA coding sequence from controls and LCHADD patients and cloned HADHAwt / HADHAmut (1528 G < C) into a retroviral expression vector (Fig. 5a). Fibroblasts from patient #5 were infected with HADHAwt, healthy control fibroblasts with the mutant form, ectopic expression was verified by qRT-PCR and mitochondrial morphology was analyzed by live cell microscopy. HADHAmut was overexpressed 5.8-fold (SD: 1.4) in wildtype fibroblasts, HADHAwt overexpression in LCHADD-derived fibroblasts was 7.4-fold (SD: 0.9). As demonstrated in Fig. 5b, the expression of a mutant allele in control fibroblasts (FB-Ctr2-mut) had no effect on mitochondrial morphology, whereas the expression of wild-type HADHA in LCHADD fibroblasts (patient #5) restored mitochondrial networks. This suggests that heterozygosity for HADHAwt is sufficient to revert the observed mitochondrial mutant phenotype.

## Discussion

The clinical treatment of LCHADD/VLCADD is mainly based on the introduction of a fat-defined diet with restriction of long-chain fat. To warrant substitution of essential long-chain fatty acids, walnut oil is given. The therapeutic intervention also includes the addition of middle chain triglycerides in the diet; eventually heptanoate (C7) is added as an anaplerotic substance. Therapy aims to compensate for the disability to metabolize long-chain fatty acids as energy substrates. Beside identification of mutations in the *HADHA* or the *ACADVL* gene in newborn screening (Supplementary Table 1) very little is known about how these mutations affect cellular metabolism and energy household. Here, for the first time we report that mutations within this mitochondrial trifunctional protein not only affect fatty acid oxidation, but lead to pronounced changes of the mitochondrial network morphology, hamper mitochondrial respiration and increase glucose consumption in patient-derived skin fibroblasts. The changed mitochondrial morphology (Fig. 1) was also reflected on a molecular level by a significantly altered ratio between the fusion protein MFN2 and its counterpart DNM1L, and a reduced pDNM1L(Ser637)/DNM1L ratio. The morphological changes correlated with reduced levels of mitochondrial respiratory complexes I and IV (Fig. 2b) and a strikingly different respiratory profile as compared to control fibroblasts: consistent with reduced complexes I and IV the fibroblasts had a reduced basal mitochondrial respiration and no spare respiratory capacity after uncoupling with FCCP (Fig. 3d). This suggests that mitochondrial respiration is already running at its limits in these cells, despite being significantly lower than in controls. Since no significant changes in overall ATP-levels were observed, our data suggest that fission-related, reduced mitochondrial efficacy in LCHADD/VLCADD patient-derived fibroblasts is largely compensated by increased glycolysis. This is in line with the observed changes in growth kinetics and glucose consumption (Fig. 3). Since mitochondrial connectivity directly relates to respiration efficacy and metabolic activity as well as ROS-generation and cell death regulation, a deregulation of mitochondrial fusion/fission has been found in several diseases^12–15^, but so far not for LCHADD or VLCADD. Especially the reduction of MFN2 or the increase of DNM1L has been associated with reduced respiratory capacity and an increase in glucose consumption for complementation of energy production^16,17^. These results strongly suggest that mutations within the *HADHA* or the *ACADVL* gene not only affect fatty acid oxidation, but exert a significant impact on general cellular metabolic activity. A similar phenomenon was described for skeletal muscle cells, where treatment with palmitate induces mitochondrial fission, DNM1L-recruitment to mitochondria and mitochondrial dysfunction^18^. Our data are also in line with a recent paper demonstrating that palmitate-induced mitochondrial-fission in rodent pancreatic beta cells relies on the activation of NOX2 (NADPH oxidase-2) and accumulation of ROS^11^. In our experiments, untreated patient-derived fibroblasts showed significantly increased steady state levels of ROS compared to untreated controls and treatment with the NOX-inhibitor Phox-I2 almost completely prevented ROS accumulation (Fig. 4a) and reverted mitochondrial fission (Fig. 4b). This clearly demonstrates that ROS accumulation depends on NOX2 activity also in LCHADD and VLCADD patient-derived fibroblasts. A possible connection between long chain fatty acids, ROS, Ca^2+^ and NOX2 activation was recently described in murine cardiomyocytes: treatment with palmitate depolarized the inner mitochondrial membrane, increased ROS, caused mitochondrial Ca^2+^ overload due to increased Ca^2+^ leakage from the endoplasmatic reticulum and activated NOX2 in a PKCalpha-dependent manner^19^. Impaired long-chain fatty acids breakdown in patient fibroblasts most likely will increase the levels of these fatty acids in mitochondria in a similar way, cause partial uncoupling of the inner mitochondrial membrane, and, possibly via Ca^2+^ release, activate PKCalpha, its downstream target NOX2 and further ROS production. Such a feed forward cycle might be responsible for permanently increased ROS levels observed in LCHADD and VLCADD fibroblasts. As a consequence of ROS accumulation (and increased Ca^2+^ levels that are also necessary for the recruitment of DNM1L to mitochondria) the mitochondrial fission machinery is activated. In contrast to palmitate-induced mitochondrial fission in muscle and beta cells, LCHADD fibroblasts do not undergo cell death, although long-term increased ROS steady state levels might lead to mitochondrial damage and dysfunction. Unlike mitochondrial fission induced by high doses of palmitate in other cell types, patient-derived LCHADD and VLCADD fibroblasts contain normal to slightly increased ATP-levels, as they apparently compensate impaired mitochondrial efficacy by increased glycolysis and elevated glucose consumption.

Our combined data may explain some clinical symptoms of LCHADD patients that are observed during episodes of fasting or exercise, when tissues with high energy consumption, like the heart muscles, run out of energy due to glucose depletion. Detailed analyses of the activity of glycolytic enzymes and mechanisms of mitochondrial ROS-regulation will uncover, how LCHADD/VLCADD mutations reprogram the mechanisms of energy generation beside long fatty acid oxidation, which in turn will lead to novel and improved treatment modalities for these rare diseases.

## Methods

### Patient fibroblasts

Skin fibroblasts from patients diagnosed with LCHADD (8 patients) or VLCADD (1 patient) were collected at the metabolic centers Vienna, Graz and Innsbruck^20^. Diagnosis was confirmed enzymatically in all but one patient (Supplementary Table 1). Skin fibroblasts from two healthy children were used as controls. The study was conducted with the approval of the ethics committee of the Medical University of Innsbruck and informed, written consent was obtained from parents of patients. Cells were cultivated in Dulbecco’s modified Eagle’s medium (Invitrogen, Carlsbad, CA, USA) containing 10% fetal bovine serum (Sigma-Aldrich, Vienna, Austria), 100 U/ml penicillin, 100 µg/ml streptomycin and 2 mM L-glutamine (Lonza, Basel, Switzerland) in 5% CO_2_, 95% relative humidity and 37 °C. All cultures were routinely tested for mycoplasma contamination using the Venor^R^ GeM-mycoplasma detection kit (Minerva Biolabs, Berlin, Germany).

### Live cell fluorescence microscopy

For live cell analyses cells were grown on collagen-coated (0.1 mg/ml) glass slides and mitochondrial morphology was analyzed by CMXRos-staining (300 nM). Images were collected using a 63x oil objective of an Axiovert200M microscope equipped with an ApoTome.2 system for structured illumination microscopy (Zeiss, Vienna, Austria)^17,21^. Images were taken at equal exposure times and processed using Axiovision software. For quantification, at least 100 cells from three independent experiments were analyzed for their mitochondrial morphology/number of branches. Mitochondria with more than 50 branches per 30 × 30 µm were classified as “small tubular”, mitochondria with more than 5 slubs/dots per 30 × 30 µm were classified as fusion defect. For ROS-measurements, the cells were grown on 8 µm chamber slides (ibidi, Martinsried, Germany) and were incubated with CM-H_2_XROS (final concentration 500 nM; Invitrogen, USA). Images were acquired with a Zeiss Axiovert200M microscope and cellular fluorescence intensity was quantified using Axiovision software (Zeiss, Vienna, Austria). Phox-I2 was purchased from Sigma-Aldrich (Vienna, Austria) and added 24 hours before microscopy.

### Immunoblot analyses

Total protein was prepared as described in Hagenbuchner *et al*.^21^. Proteins were separated by SDS-PAGE and blotted on nitrocellulose membrane (GE Healthcare, Chalfot, UK). After blocking, membranes were incubated with primary antibodies against MFN1, MFN2, DNM1L, and OXPHOS (Abcam, Cambridge, UK), pDNM1L(Ser637) and α-Tubulin (Cell Signaling Technology Inc., Boston, USA), and GAPDH (Acris, Herford, Germany) washed and incubated with horseradish-peroxidase conjugated secondary antibody (GE Healthcare, UK). The immunoblots were developed by enhanced chemiluminescence (GE Healthcare, Chalfont, UK) according to manufacturer’s instructions and analyzed using an AutoChemi detection system (UVP, Cambridge, UK). Densitometry was performed using Labworks software version 4.5 (UVP, UK). Uncropped blots of three independently generated panels are shown in Supplemental Fig. 1.

### Glucose determination

Glucose consumption was measured after cultivation of identical cell numbers for 72 hours by colorimetry using the Glucose Assay Kit (BioVision, Mountain View, USA) according to the manufacturer’s instruction^17^. For quantification, levels were normalized to the cell number at the endpoint.

### ATP Assay

Identical cell numbers of fibroblasts were gown in white 96 well plates with a clear bottom for 72 hours. ATP amount was quantified by luminescence detection using CellTiterGlow® Luminscence assay (Promega, Mannheim, Germany).

### Respirometry

8.000 fibroblasts/well were seeded in Seahorse XFp cell culture miniplates 24 hours before measurements. Analyses of respiration and inhibition/uncoupling of respiratory chain complexes were done using Agilent Seahorse XFp Cell mito stress test kit (Agilent Technologies, Santa Clara, CA, USA) in a Seahorse XFp System (Agilent Technologies, Santa Clara, CA, USA) according to manufacturer’s instructions.

### Statistics

Statistical significance of differences between control cells and patients were assessed using Student’s unpaired t-test. Each patient was calculated to both individual controls and assessed as significantly different, only when both analyses were significantly different. All statistical analyses and *P*-values were calculated using the GraphPadPrism 7.0.

### Cloning of HADHA and generation of transgenic cell lines

The full coding region of HADHA was amplified from cDNA prepared from healthy control fibroblasts (Ctr2) and LCHADD patient #5 by PCR using the primers HADHA_fwd (TATAGTCGACTCAAGATGGTGGCCTGCC) and the HADHA_rev (TATAGCGGCCGCTGACTGAGCGAGGCATGAGG). The PCR product was then cloned into the Sal1-Not1 sites of pLIB-MCS2-iresNeo^22^ generating pLIB-HADHAwt-iresNeo and pLIB-HADHAmut-iresNeo, respectively. The HADHA open reading frames in these vectors were sequenced by Microsynth (Balgach, Switzerland). For infection of cells retroviruses were produced as described before^17,21^ and used for infecting the healthy control cell line (FB-Ctr2) for generation of FB-Ctr2-HADHAmut or the patient cell line FB-LCHADD#5 generating FB-LCHADD#5-HADHAwt.

### Quantitative RT-PCR

To quantify HADHA mRNA expression levels in retrovirally infected cells we designed real-time RT-PCR assays, using GAPDH as reference gene. Total RNA was isolated from 1 × 10^6^ cells using Tri Reagent (Sigma-Aldrich, Vienna, Austria) according to the manufacturer’s instructions. Complementary DNA was synthesized from 1 µg of total RNA using the RevertAid^TM^ First Strand H minus cDNA Synthesis Kit (Thermo Scientific, Sankt Leon-Rot, Germany). The oligonucleotides to amplify cDNA fragments were HADHA_RT_fwd (GCCCATGATGTCTGAAGTCATCC) and HADHA_RT_rev (CGCTACATCCACACCAACTTCATC) synthesized by Microsynth AG (Balgach, Switzerland). After normalization on GAPDH expression, regulation was calculated between ectopic HADHA-expressing cells and controls. Controls are set as 100% expression.

### Data availability and confirmation

All data generated or analyzed during this study are included in this published article. We confirm that all experiments were performed in accordance with relevant guidelines and regulations.

## Electronic supplementary material

Supplementary Figures

Supplementary Table
