# Supplementary material for: Very long-/ and long Chain-3-Hydroxy Acyl CoA Dehydrogenase Deficiency correlates with deregulation of the mitochondrial fusion/fission machinery
Source: Sci Rep. 2018 Feb 19;8:3254. doi: 10.1038/s41598-018-21519-2 (PMC5818531; doi:10.1038/s41598-018-21519-2)
Supplement: Supplementary file 1 — Supplementary Figures [file 41598_2018_21519_MOESM1_ESM.pdf]

Supplementary Information

Very long-/ and long Chain-3-Hydroxy Acyl CoA Dehydrogenase Deficiency correlates with deregulation of the mitochondrial fusion/fission machinery

Judith Hagenbuchner[1], Sabine Scholl-Bürgi[2], Daniela Karall[2]*, and Michael J. Ausserlechner[2]*
[1]Departments of Pediatrics II and [2]Pediatrics I, Medical University Innsbruck, Innsbruck, Austria
*corresponding authors

Supplemental Figure 1: Original uncropped blots

A) Analyses of Fusion/Fission proteins in LCHADD/VLCADD patients
PANEL 1:

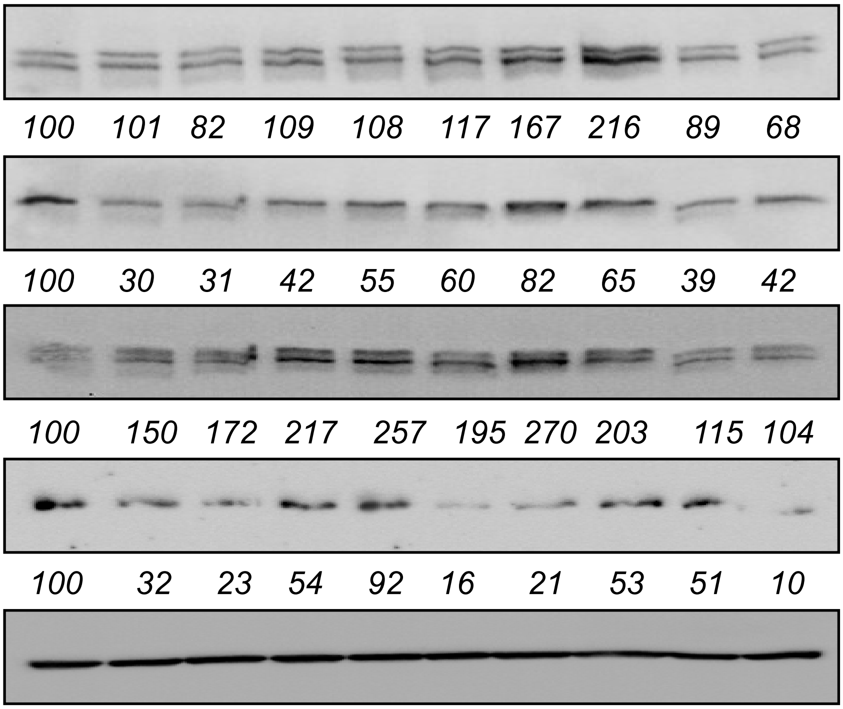

	Ctr	LCHADD								VLCADD
	1	1	2	3	4	5	6	7	8	1
MFN1 % fold over control	*100*	*101*	*82*	*109*	*108*	*117*	*167*	*216*	*89*	*68*
MFN2 % fold over control	*100*	*30*	*31*	*42*	*55*	*60*	*82*	*65*	*39*	*42*
DNM1L % fold over control	*100*	*150*	*172*	*217*	*257*	*195*	*270*	*203*	*115*	*104*
pDNM1L(Ser637) % fold over control	*100*	*32*	*23*	*54*	*92*	*16*	*21*	*53*	*51*	*10*
% MFN2/DNM1L (fold over control)	***100***	***20***	***18***	***19***	***21***	***31***	***30***	***32***	***34***	***40***
% pDNM1L/DNM1L (fold over control)	***100***	***21***	***13***	***25***	***36***	***8***	***8***	***26***	***44***	***10***

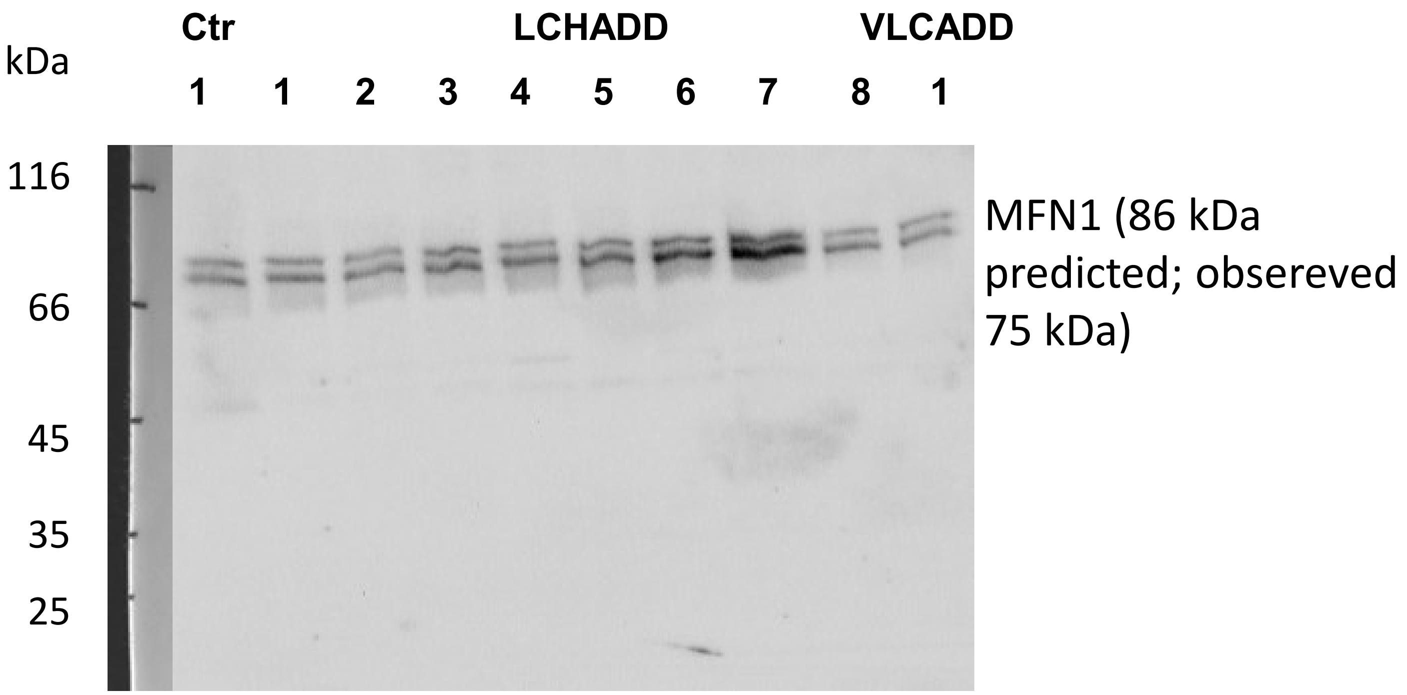
Ctr
LCHADD
VLCADD
kDa
1 1 2 3 4 5 6 7 8 1
116
66
45
35
25
MFN1 (86 kDa predicted; obsereved 75 kDa)

MFN2

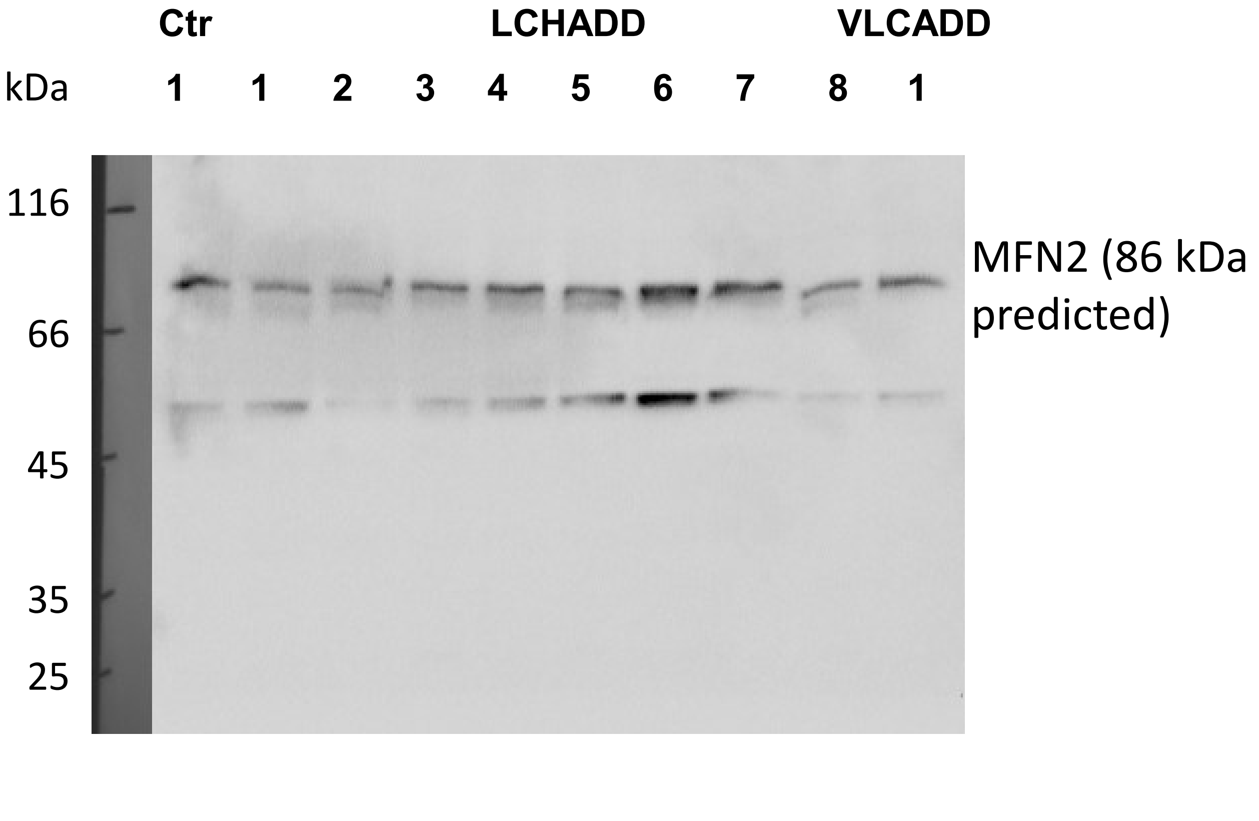
Ctr
LCHADD
VLCADD
kDa
1 1 2 3 4 5 6 7 8 1
116
66
45
35
25
MFN2 (86 kDa predicted)

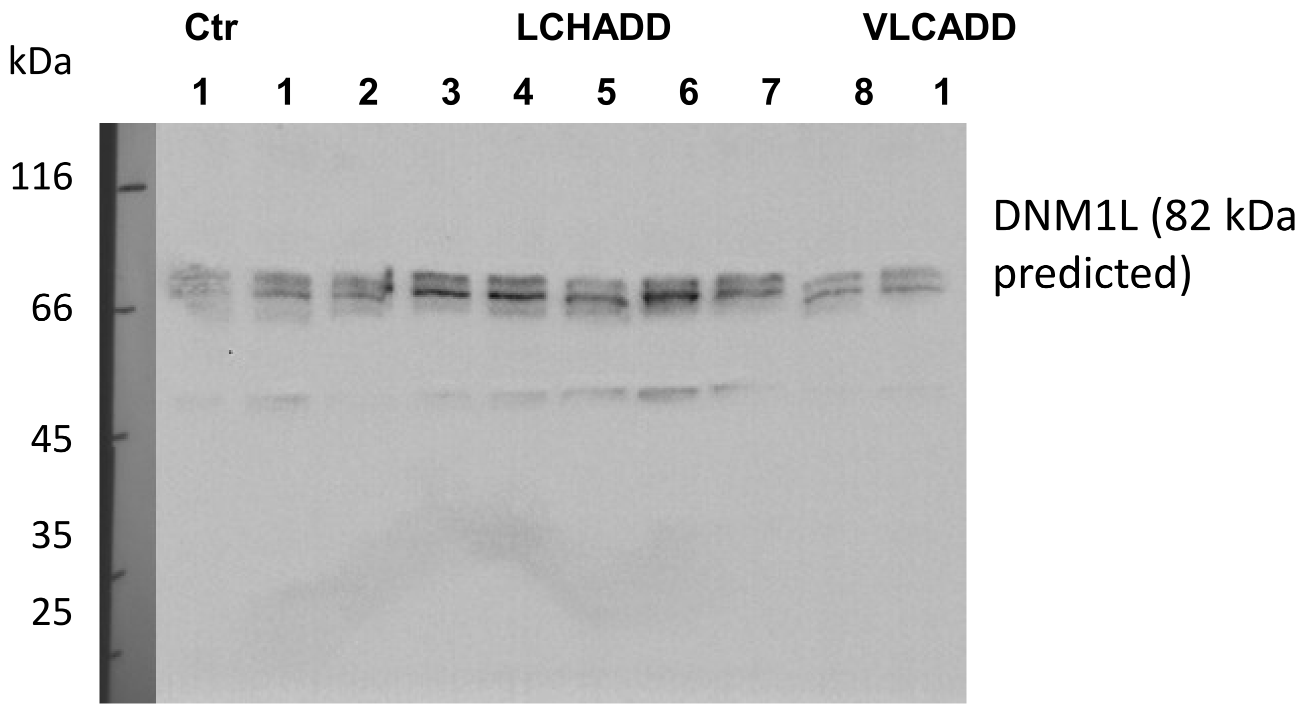
Ctr
LCHADD
VLCADD
kDa
1 1 2 3 4 5 6 7 8 1
116
66
45
35
25
DNM1L (82 kDa predicted)

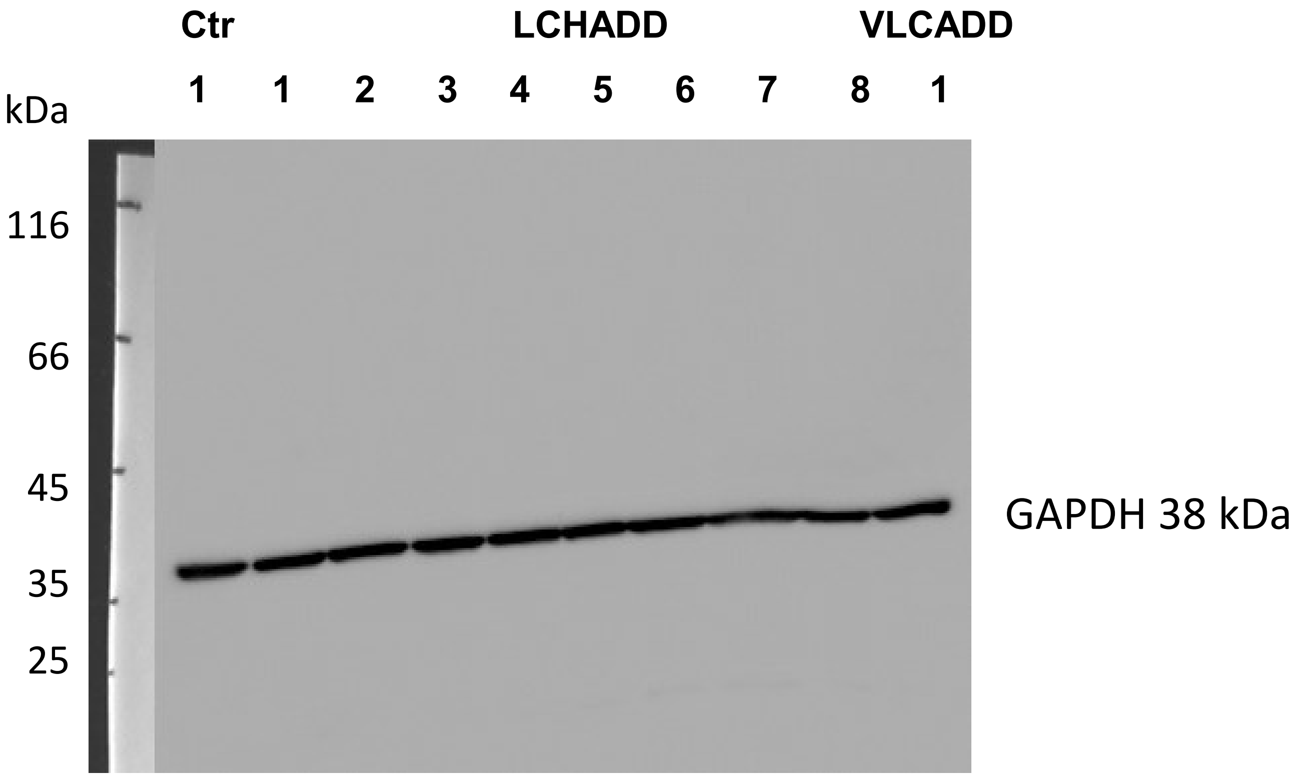
Ctr
LCHADD
VLCADD
kDa
1 1 2 3 4 5 6 7 8 1
116
66
45
35
25
GAPDH 38 kDa

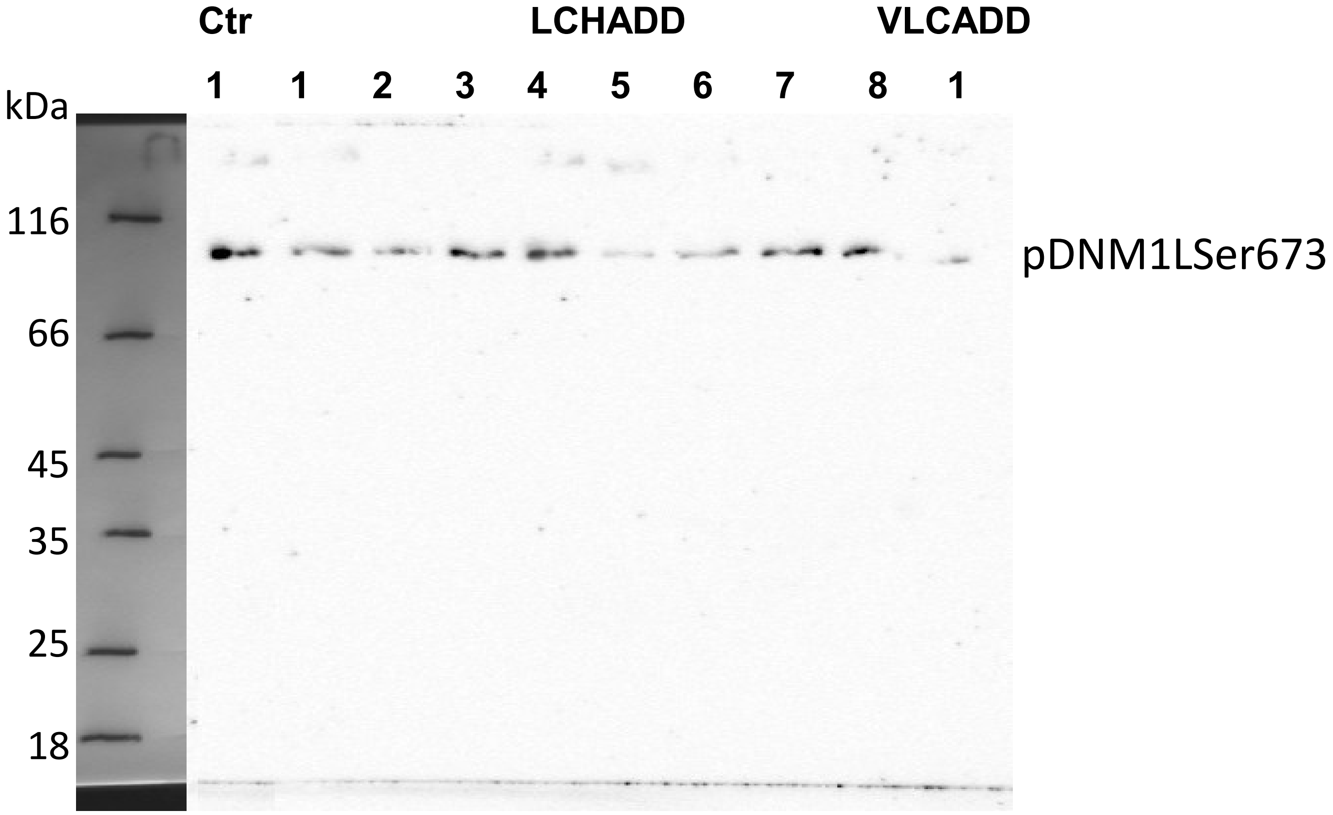
Ctr
LCHADD
VLCADD
1 1 2 3 4 5 6 7 8 1
kDa
116
66
45
35
25
18
pDNM1LSer673

Analyses of Fusion/Fission proteins in LCHADD/VLCADD patients
PANEL 2:

Ctr	LCHADD								VLCADD
1	1	2	3	4	5	6	7	8	1

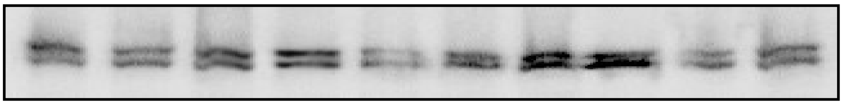

MFN1

100	*83*	*97*	*113*	*56*	*87*	*120*	*140*	*66*	*89*	*% fold over control*

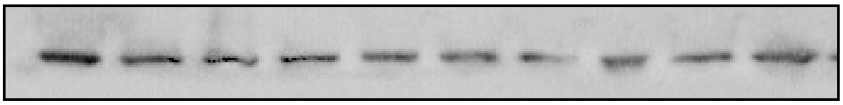

MFN2

100	*57*	*56*	*53*	*47*	*44*	*37*	*47*	*45*	*39*	*% fold over control*

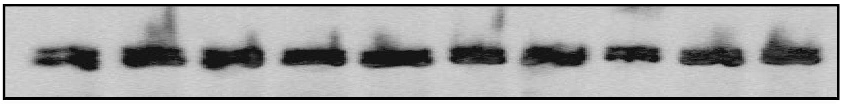

DNM1L

100	*138*	*121*	*111*	*129*	*101*	*138*	*90*	*120*	*96*	*% fold over control*

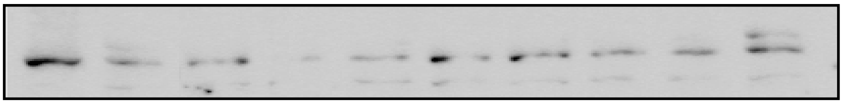

pDNM1L(Ser637)

100	*42*	*37*	*32*	*25*	*51*	*58*	*41*	*46*	*57*	*% fold over control*

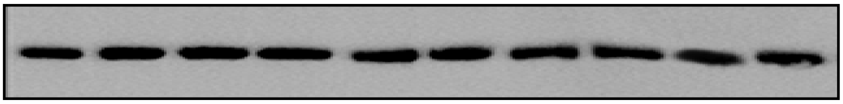

Tubulin

100	***41***	***46***	***48***	***37***	***44***	***27***	***53***	***37***	***40***	***% MFN2/DNM1L (fold over control)***
100	***30***	***31***	***29***	***19***	***51***	***42***	***46***	***38***	***59***	***% pDNM1L/DNM1L (fold over control)***

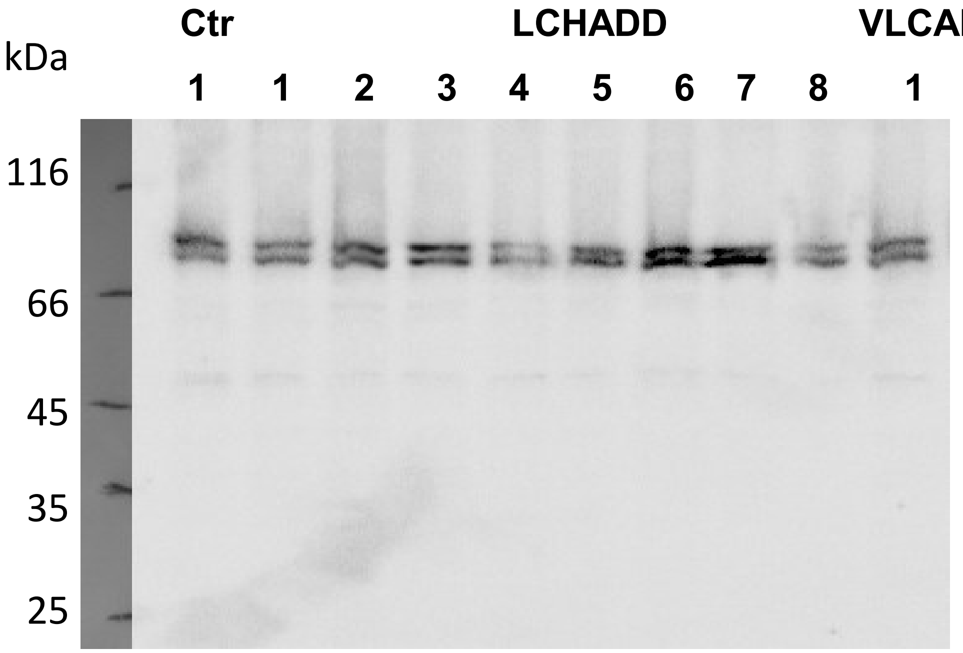

MFN1 (86 kDa predicted; obsereved 75 kDa)

MFN2

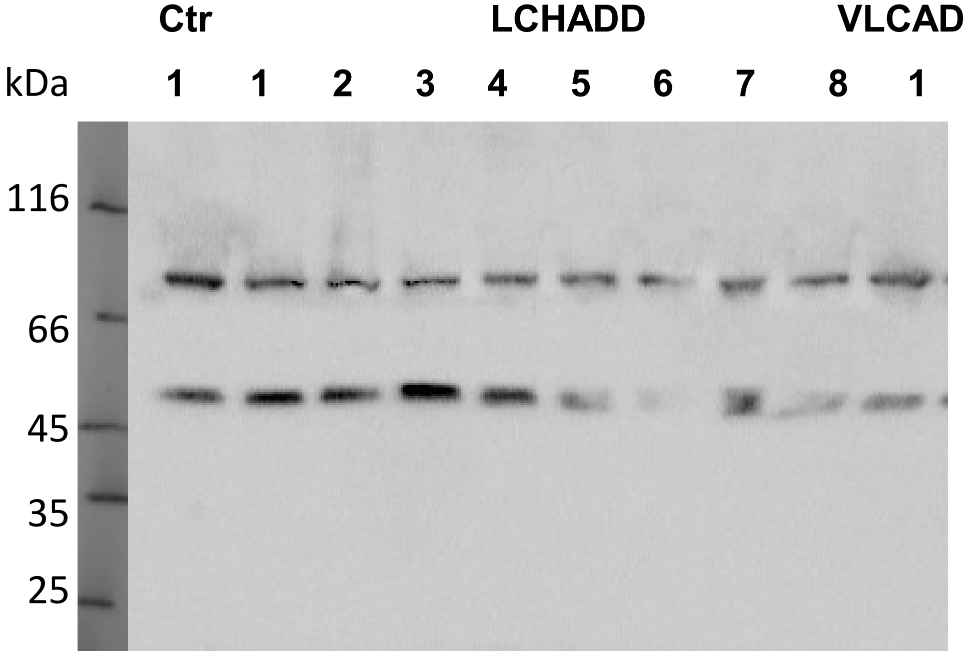

MFN2 (86 kDa predicted)

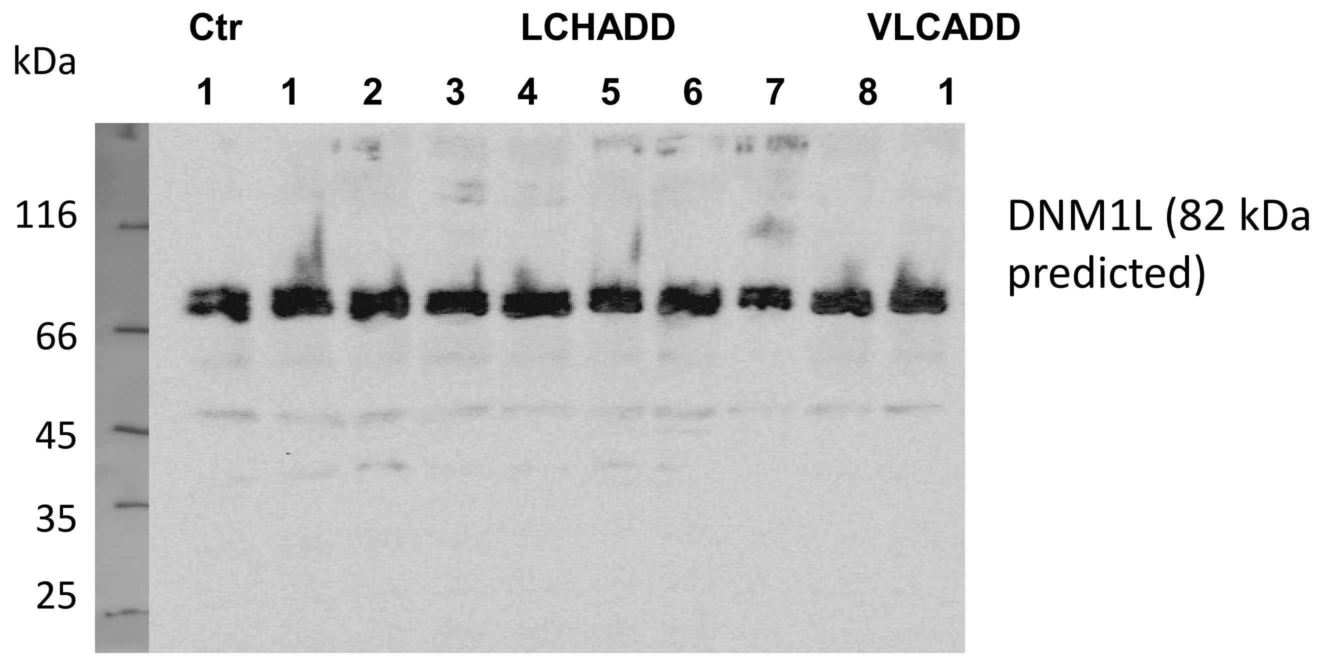
Ctr
LCHADD
VLCADD
kDa
1 1 2 3 4 5 6 7 8 1
116
66
45
35
25
DNM1L (82 kDa predicted)

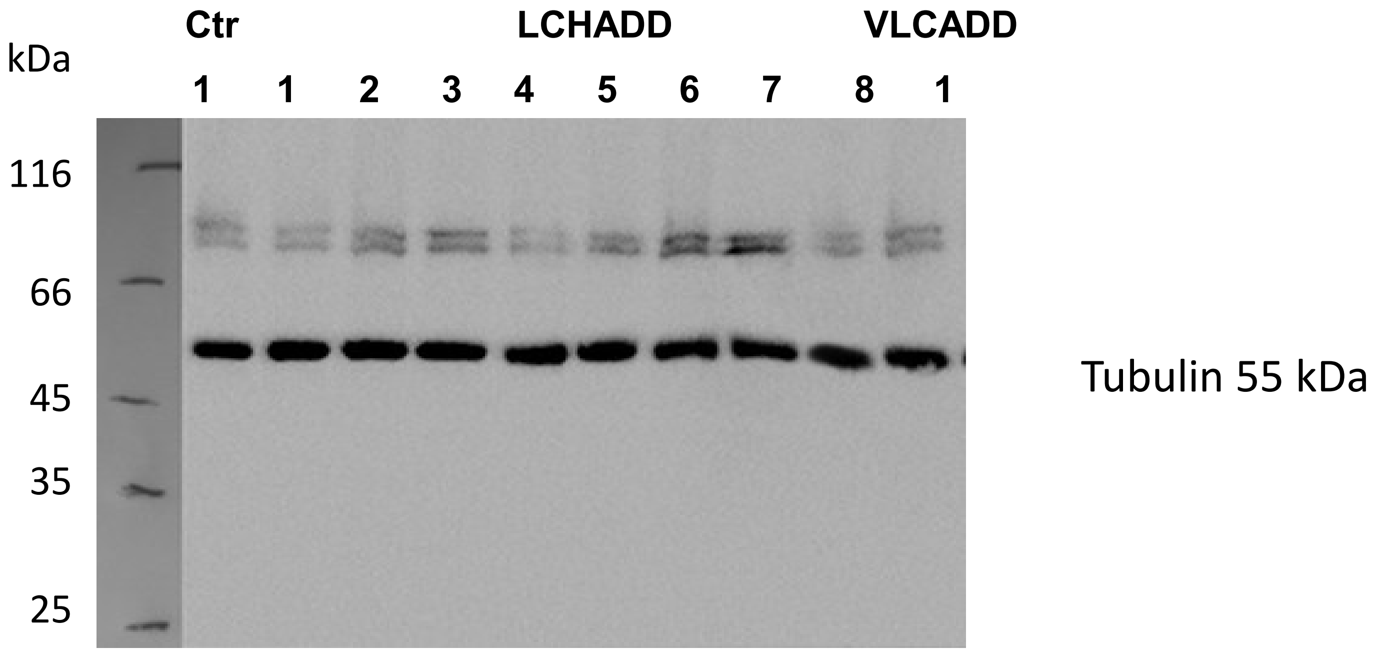
Ctr
LCHADD
VLCADD
kDa
1 1 2 3 4 5 6 7 8 1
116
66
45
35
25
Tubulin 55 kDa

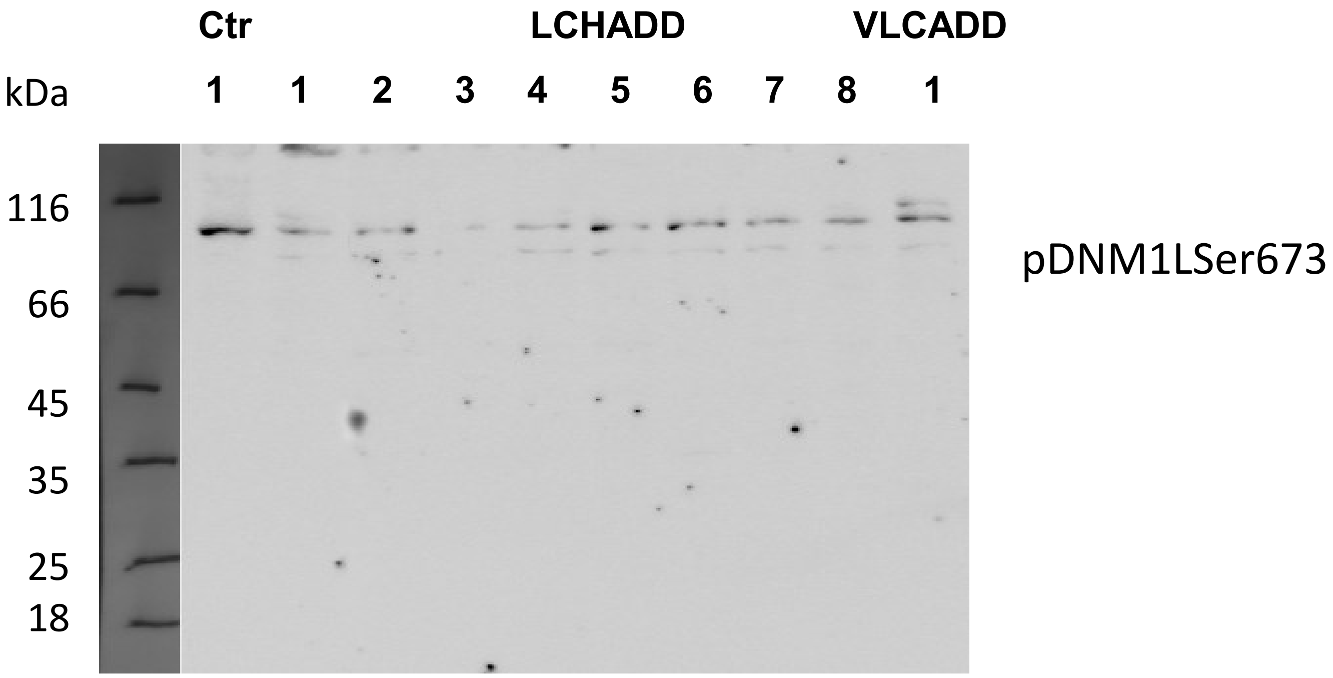
Ctr
LCHADD
VLCADD
kDa
1 1 2 3 4 5 6 7 8 1
116
66
45
35
25
18
pDNM1LSer673

Analyses of Fusion/Fission proteins in LCHADD/VLCADD patients
PANEL 3:

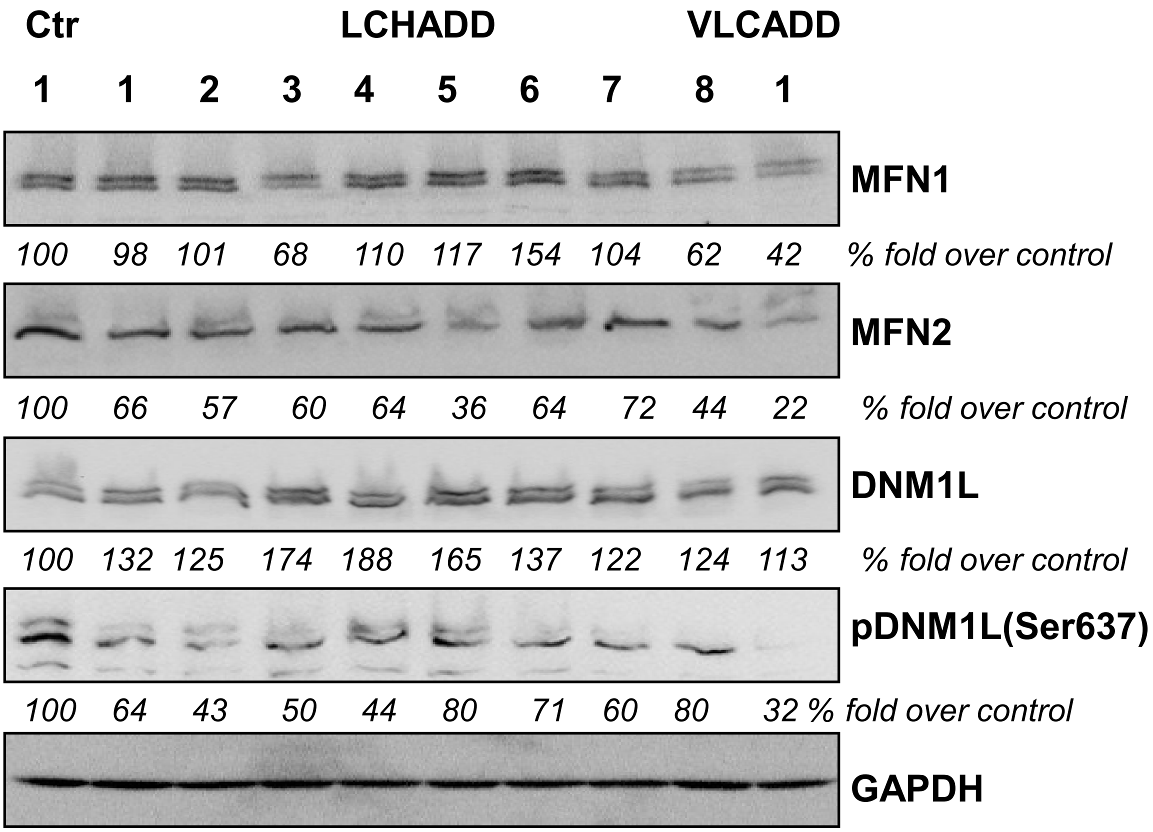

100	***50***	***45***	***35***	***29***	***22***	***47***	***60***	***36***	***20***	***% MFN2/DNM1L (fold over control)***
100	***48***	***34***	***29***	***24***	***48***	***52***	***49***	***64***	***28***	***% pDNM1L/DNM1L (fold over control)***

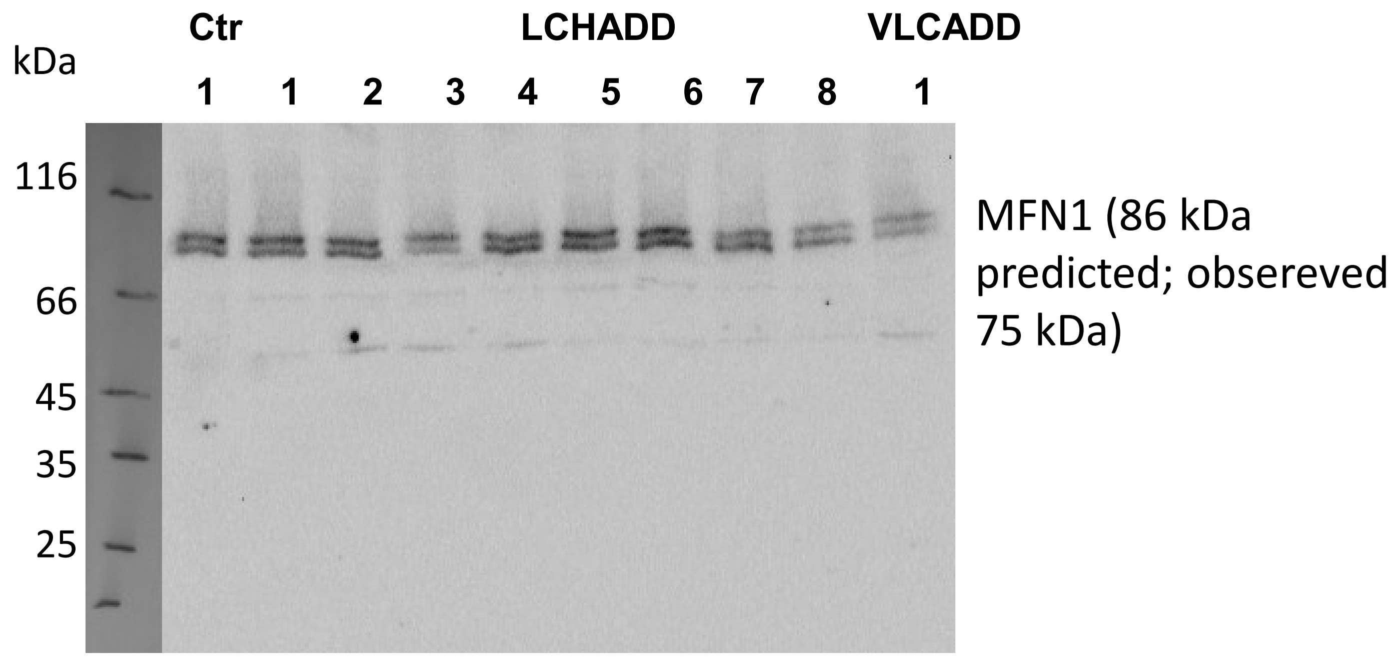
Ctr
LCHADD
VLCADD
kDa
1 1 2 3 4 5 6 7 8 1
116
66
45
35
25
MFN1 (86 kDa predicted; obsereved 75 kDa)

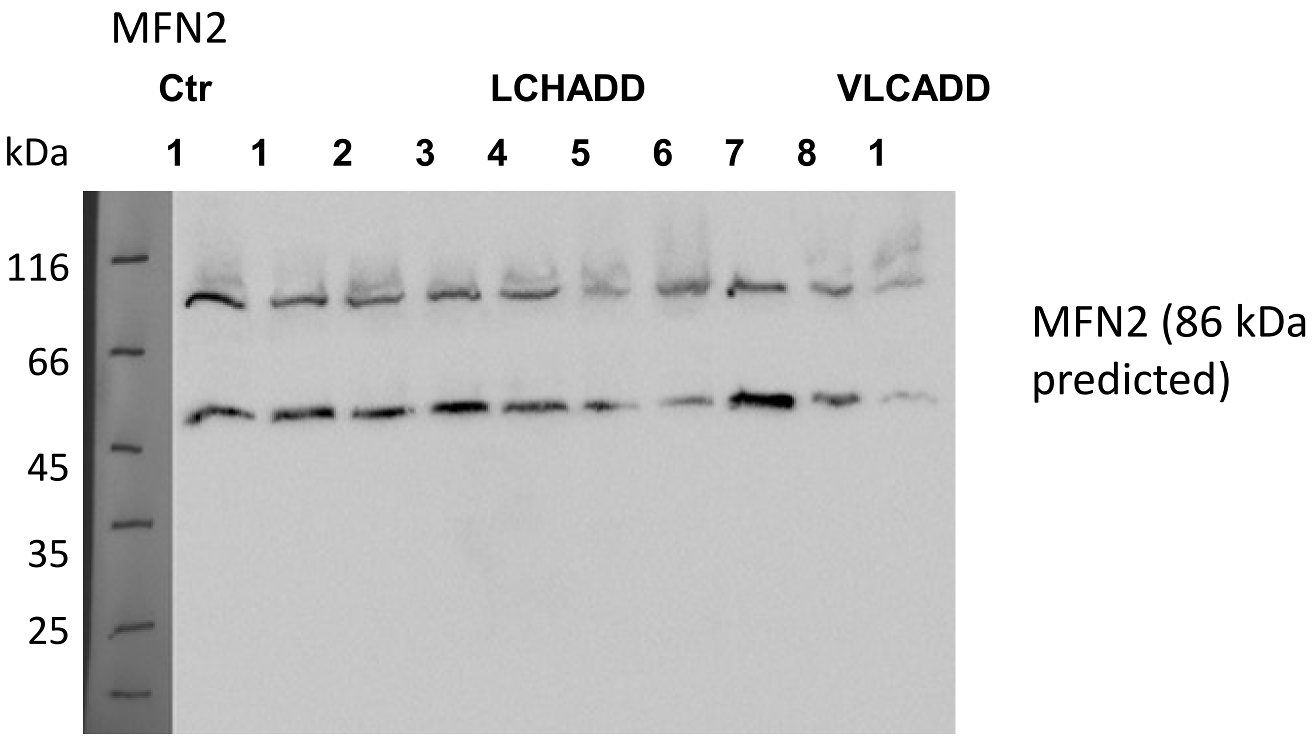
MFN2
Ctr
LCHADD
VLCADD
kDa
1 1 2 3 4 5 6 7 8 1
116
66
45
35
25
MFN2 (86 kDa predicted)

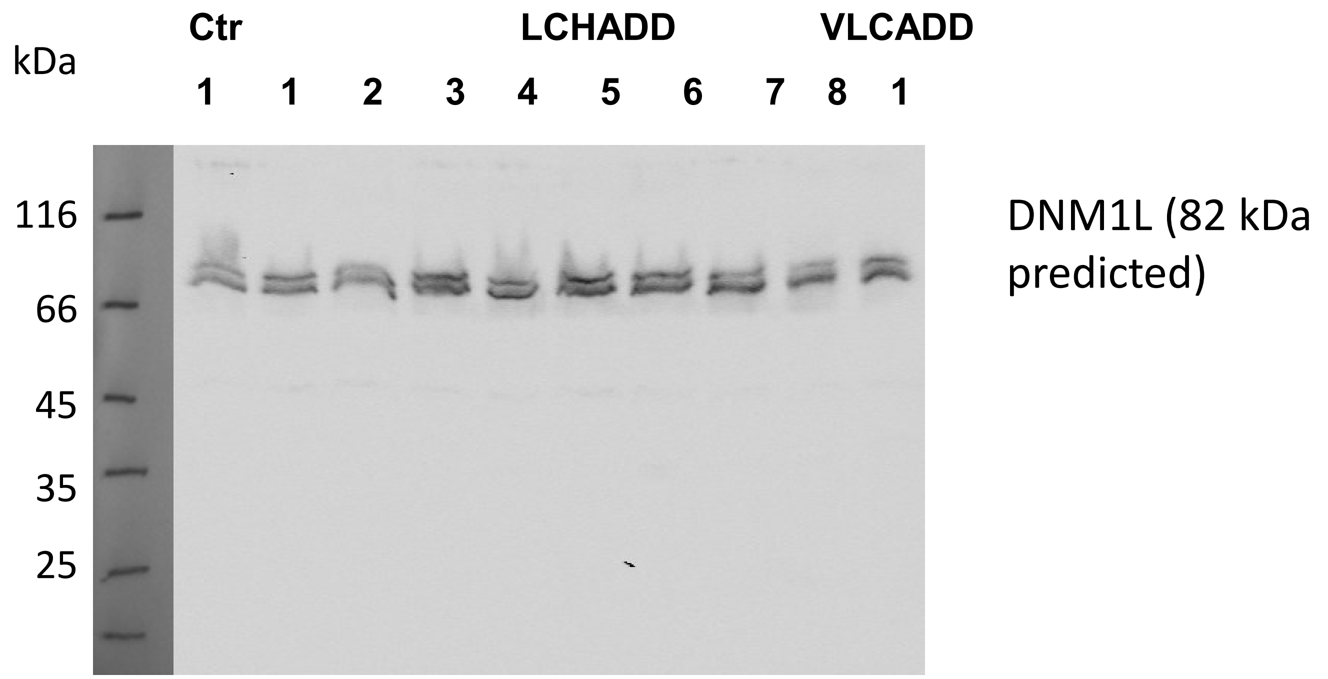
Ctr
LCHADD
VLCADD
kDa
1 1 2 3 4 5 6 7 8 1
116
66
45
35
25
DNM1L (82 kDa predicted)

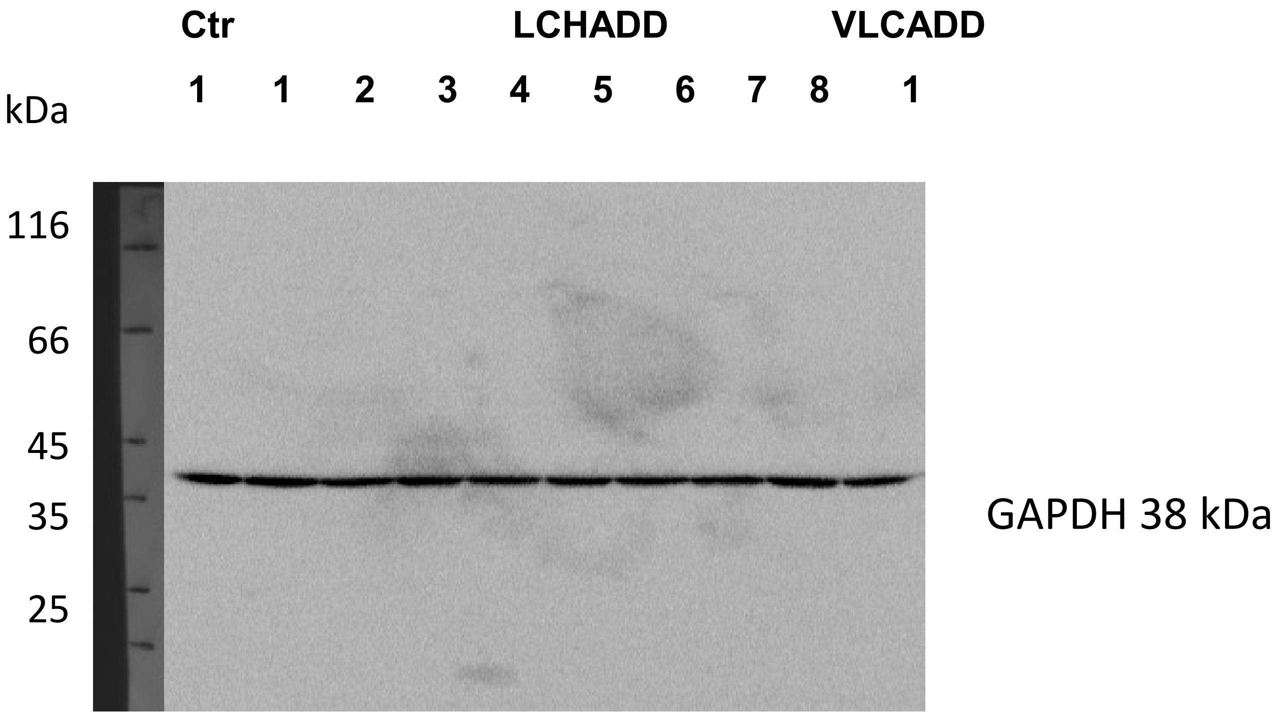
Ctr
LCHADD
VLCADD
1 1 2 3 4 5 6 7 8 1
kDa
116
66
45
35
25
GAPDH 38 kDa

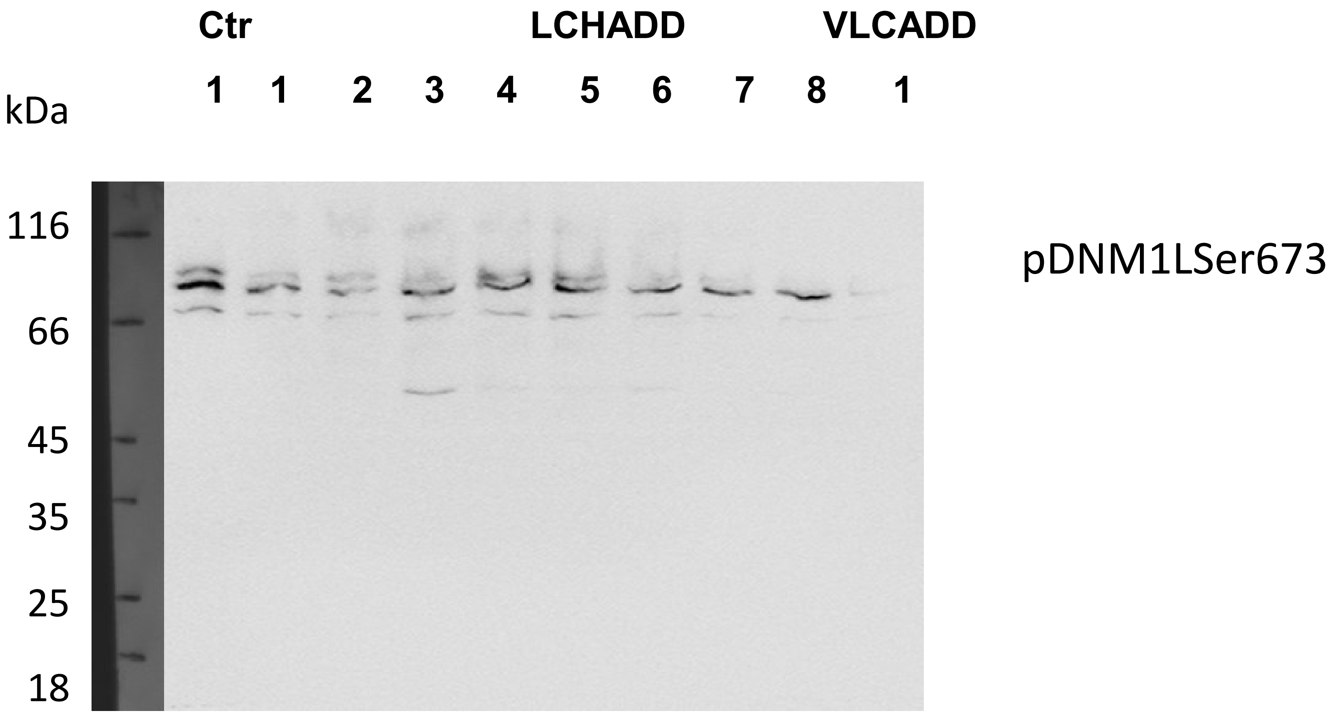
Ctr
LCHADD
VLCADD
1
1
2
3
4
5
6
7
8
1
kDa
116
66
45
35
25
18
pDNM1LSer673

B) Analyses of OXPHOS in LCHADD/VLCADD patients:

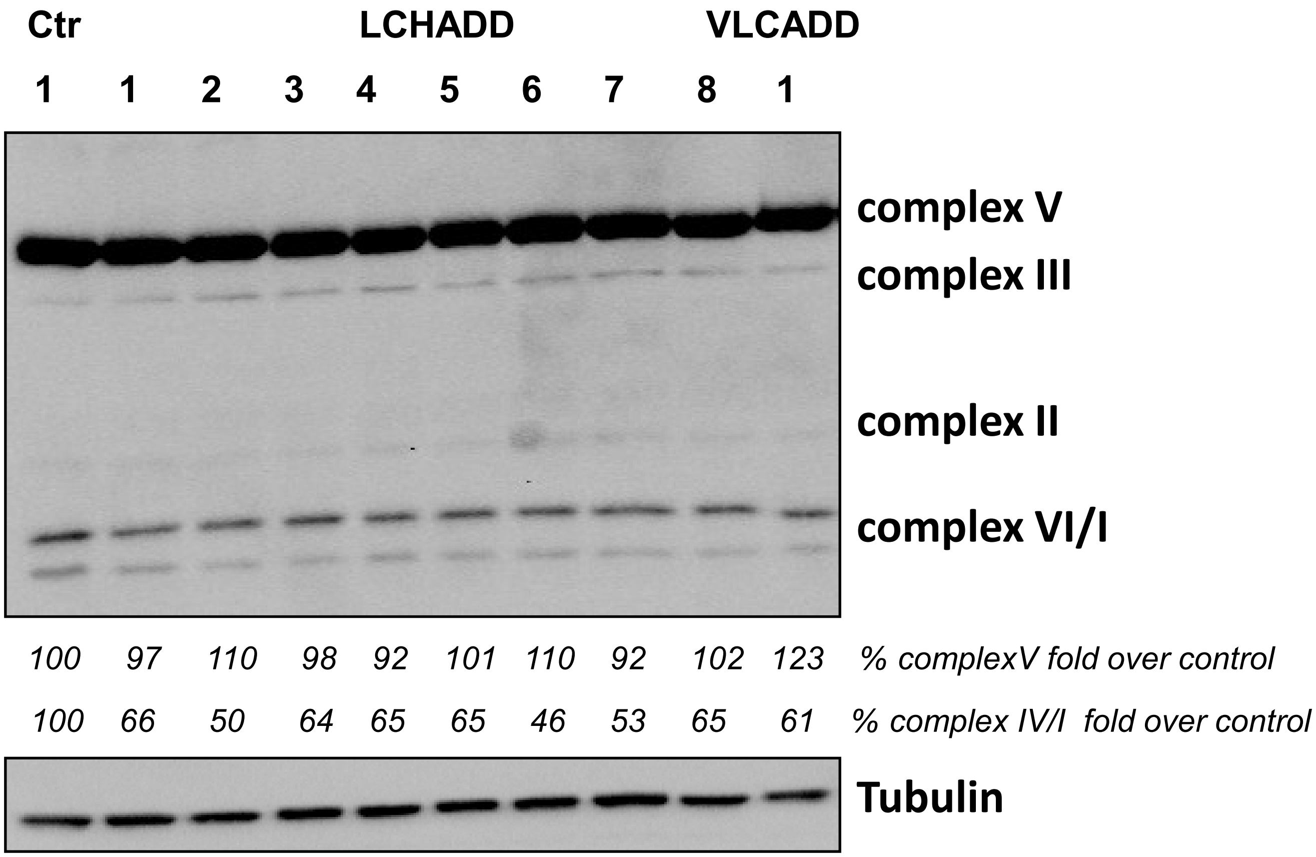

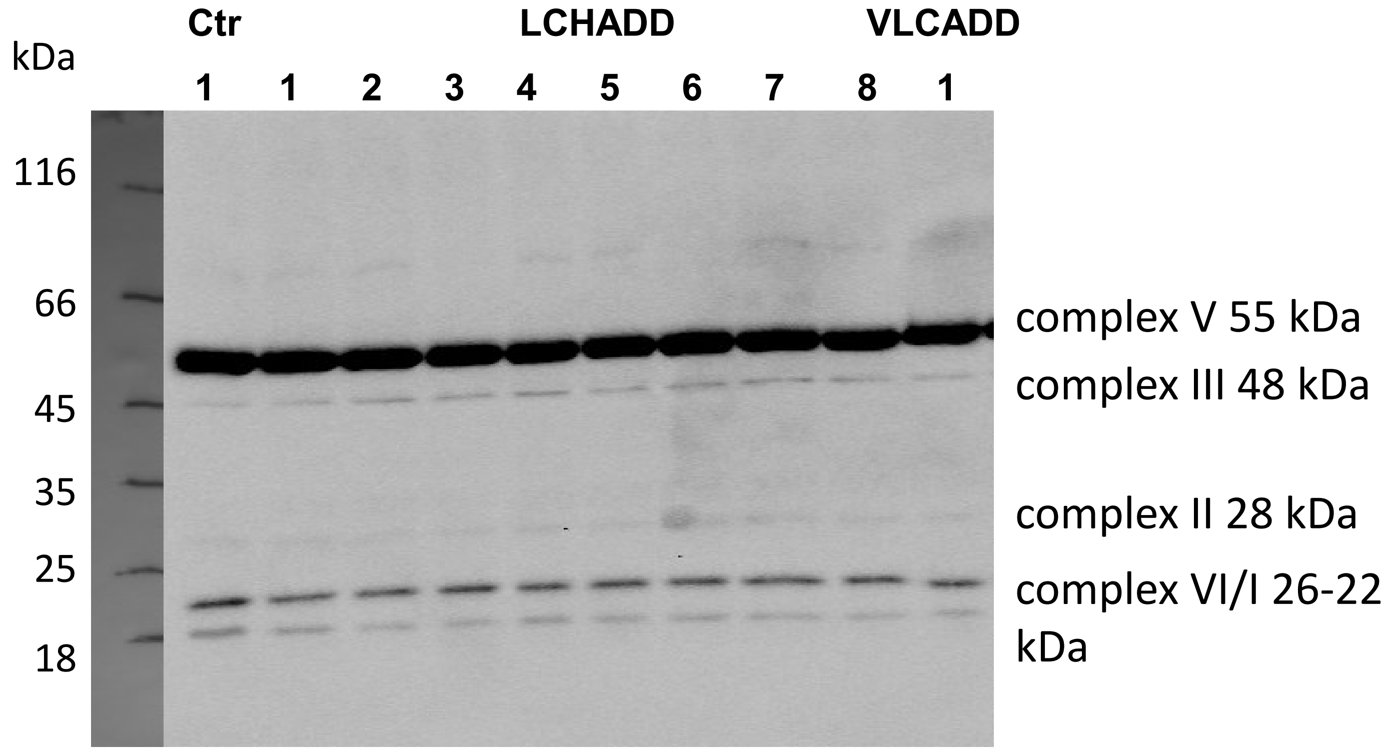
Ctr
LCHADD
VLCADD
kDa
1 1 2 3 4 5 6 7 8 1
116
66
45
35
25
18
complex V 55 kDa
complex III 48 kDa
complex II 28 kDa
complex VI/I 26-22 kDa

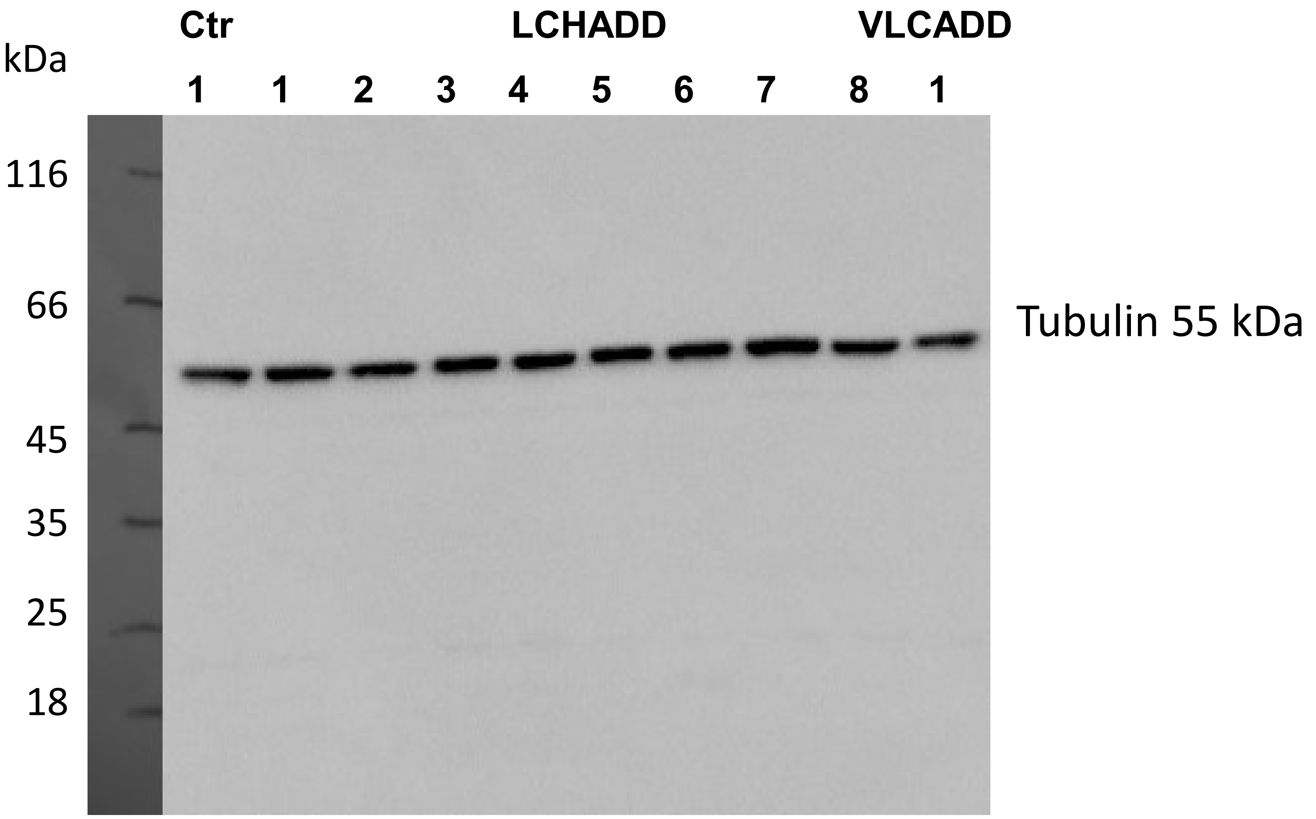
Ctr
LCHADD
VLCADD
kDa
1 1 2 3 4 5 6 7 8 1
116
66
45
35
25
18
Tubulin 55 kDa

Analyses of OXPHOS in LCHADD/VLCADD patients PANEL 2:

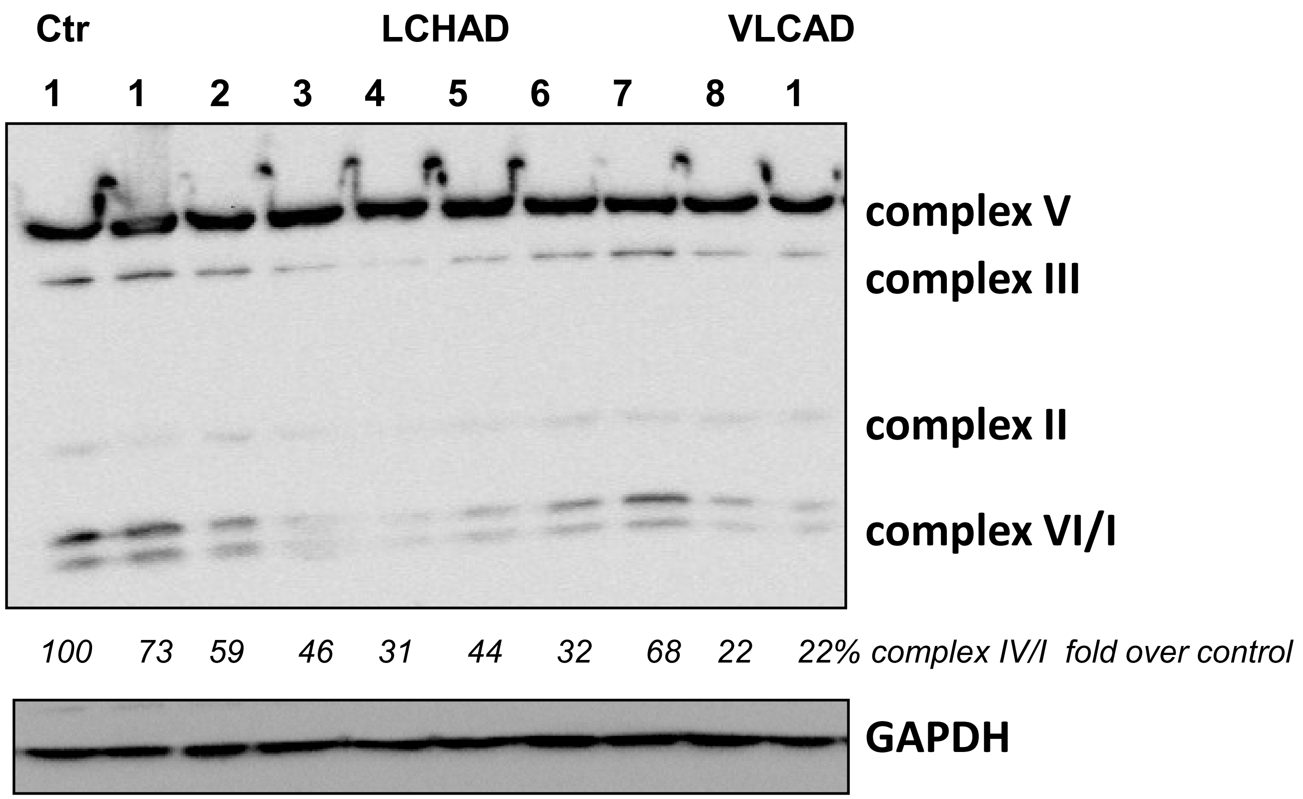

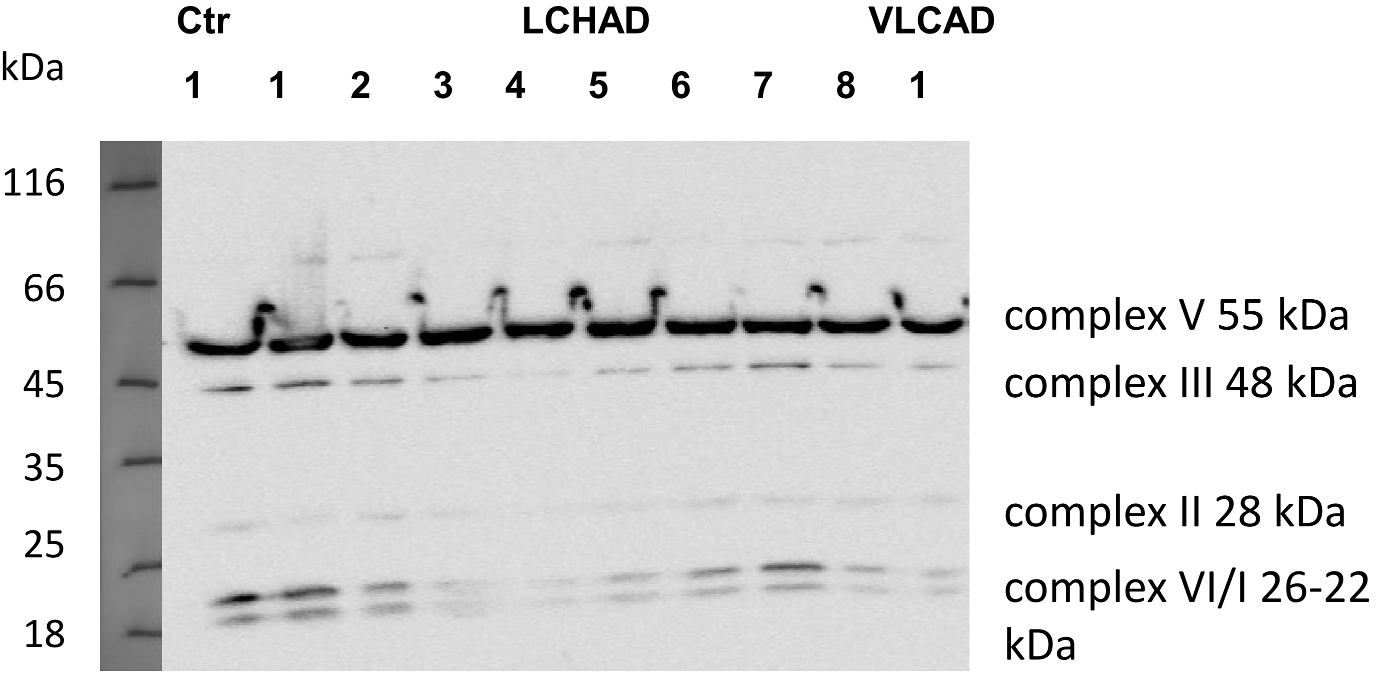
Ctr
LCHAD
VLCAD
kDa
1 1 2 3 4 5 6 7 8 1
116
66
45
35
25
18
complex V 55 kDa
complex III 48 kDa
complex II 28 kDa
complex VI/I 26-22 kDa

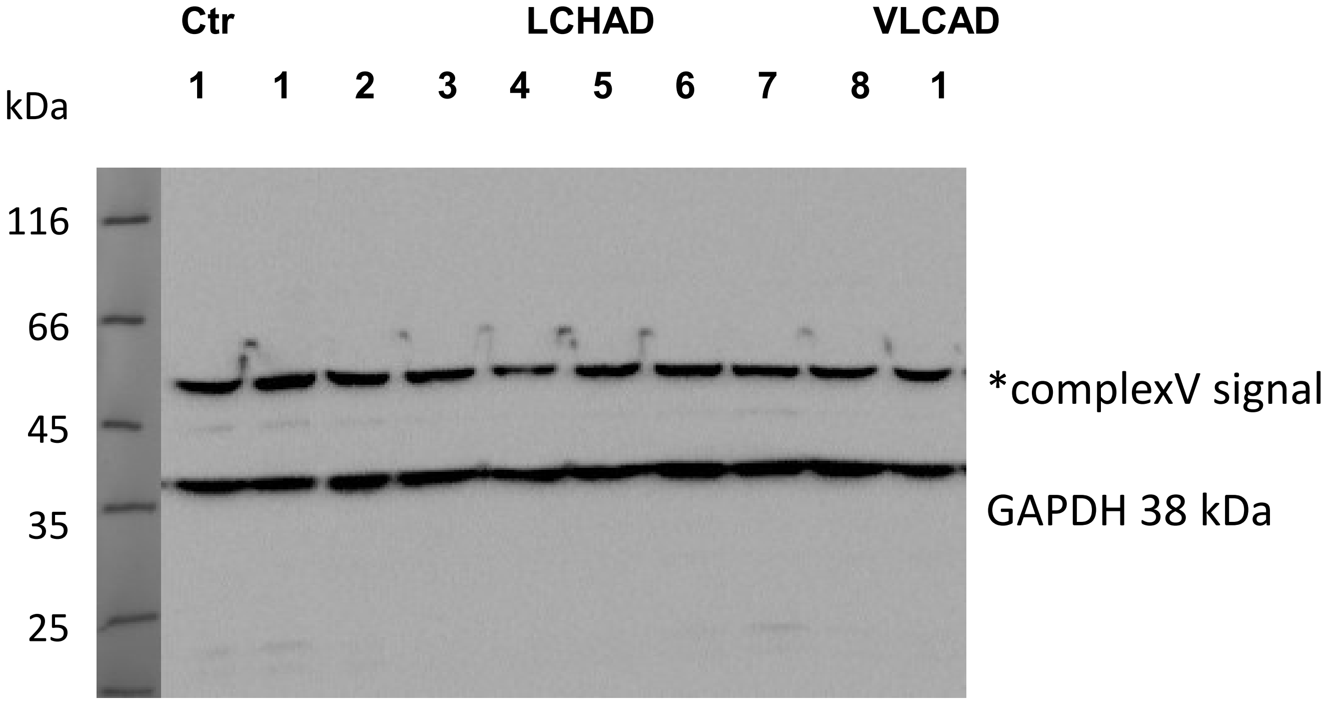
Ctr
LCHAD
VLCAD
kDa
1 1 2 3 4 5 6 7 8 1
116
66
45
35
25
*complexV signal
GAPDH 38 kDa

Analyses of OXPHOS in LCHADD/VLCADD patients PANEL 3:

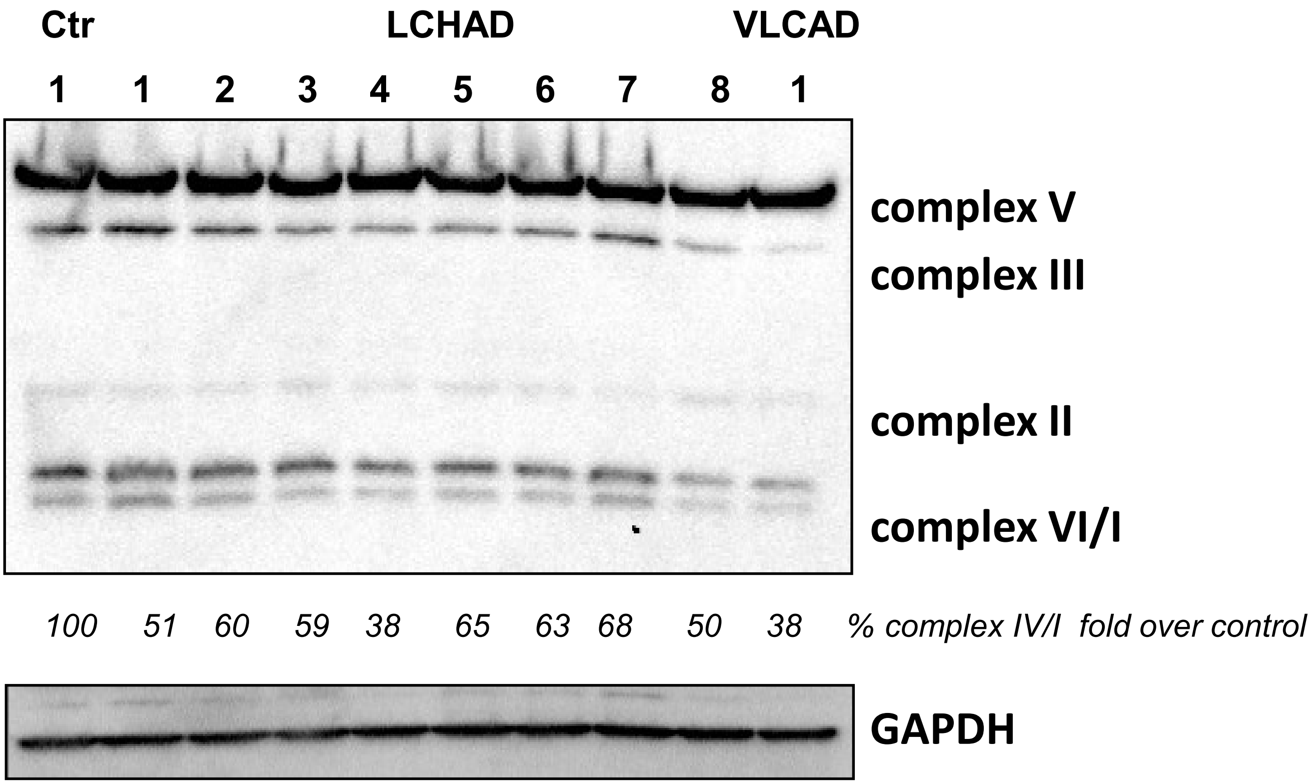

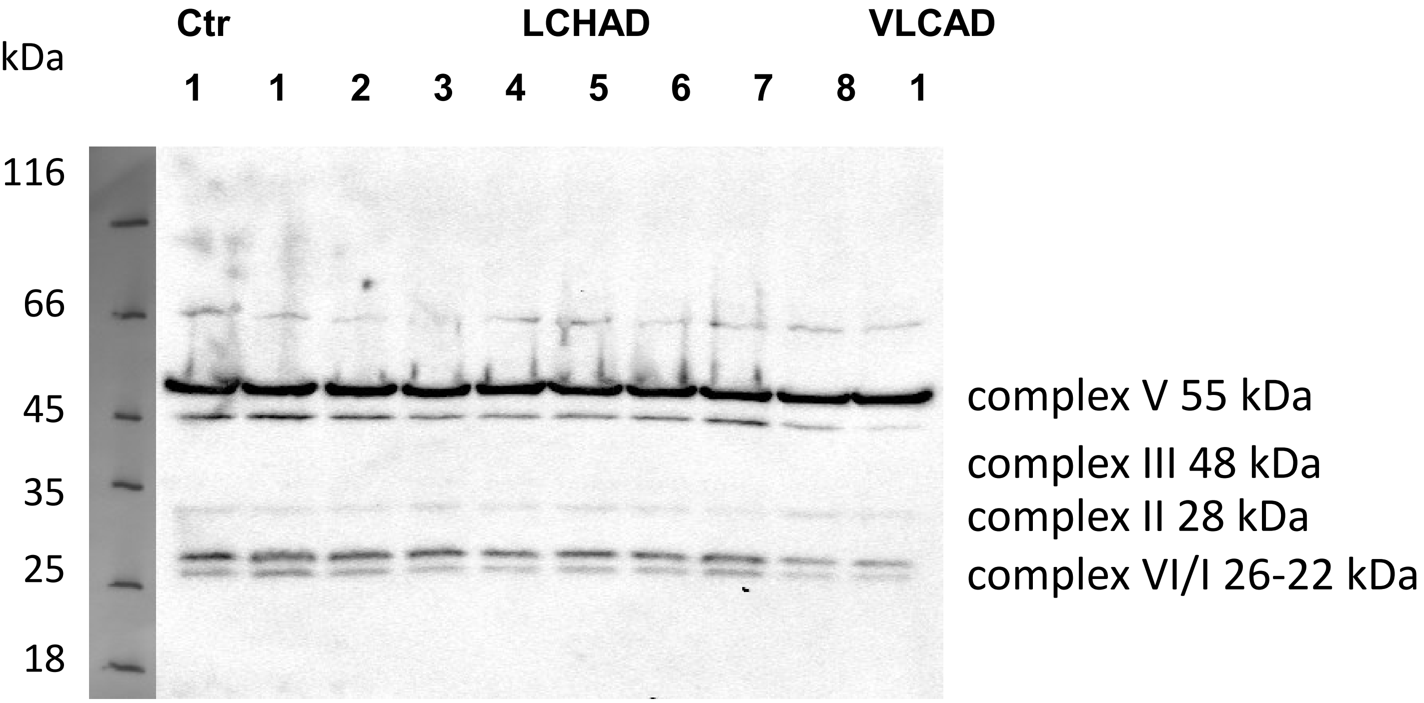

Ctr
LCHAD
VLCAD
kDa
1 1 2 3 4 5 6 7 8 1
116
66
45
35
25
18
complex V 55 kDa
complex III 48 kDa
complex II 28 kDa
complex VI/I 26-22 kDa

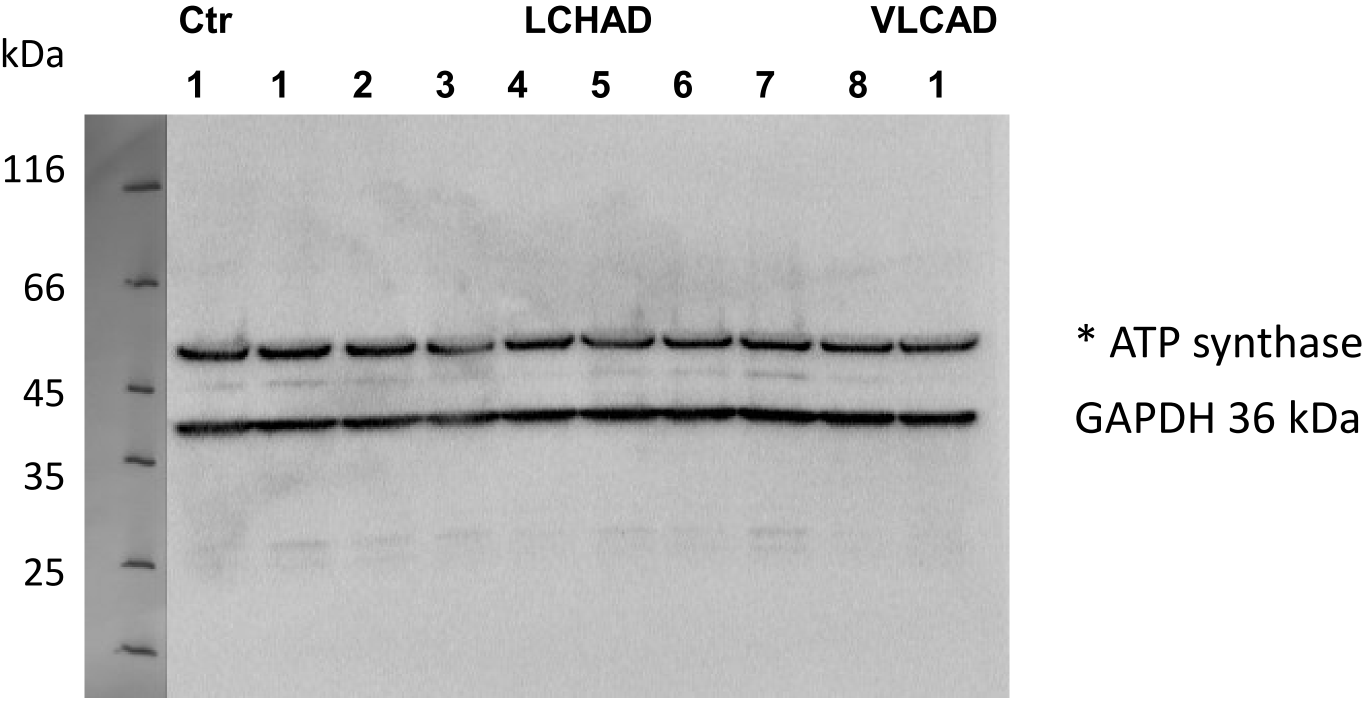

Ctr
LCHAD
VLCAD
kDa
1 1 2 3 4 5 6 7 8 1
116
66
45
35
25
* ATP synthase
GAPDH 36 kDa
